# Cingulin and actin mediate midbody-dependent apical lumen formation during polarization of epithelial cells

**DOI:** 10.1038/ncomms12426

**Published:** 2016-08-03

**Authors:** Anthony J. Mangan, Daniel V. Sietsema, Dongying Li, Jeffrey K. Moore, Sandra Citi, Rytis Prekeris

**Affiliations:** 1 Department of Cell and Developmental Biology, School of Medicine, Anschutz Medical Campus, University of Colorado Denver, Aurora, Colorado 80045, USA; 2 Cell Biology Department, University of Geneva, CH-1211 GENEVA 4, Switzerland

## Abstract

Coordinated polarization of epithelial cells is a key step during morphogenesis that leads to the formation of an apical lumen. Rab11 and its interacting protein FIP5 are necessary for the targeting of apical endosomes to the midbody and apical membrane initiation site (AMIS) during lumenogenesis. However, the machinery that mediates AMIS establishment and FIP5-endosome targeting remains unknown. Here we identify a FIP5-interacting protein, Cingulin, which localizes to the AMIS and functions as a tether mediating FIP5-endosome targeting. We analysed the machinery mediating AMIS recruitment to the midbody and determined that both branched actin and microtubules are required for establishing the site of the nascent lumen. We demonstrate that the Rac1-WAVE/Scar complex mediates Cingulin recruitment to the AMIS by inducing branched actin formation, and that Cingulin directly binds to microtubule C-terminal tails through electrostatic interactions. We propose a new mechanism for apical endosome targeting and AMIS formation around the midbody during epithelial lumenogenesis.

## Abstract (web summary)

Polarisation of epithelial cells causes lumen formation, which is mediated by apical membrane initiation site (AMIS) and FIP5, but how this is regulated is unclear. Here, the authors identify cingulin as a FIP-5 interacting protein, recruiting the Rac1-WAVE/Scar complex to the AMIS and branched actin formation.

## Graphical abstract

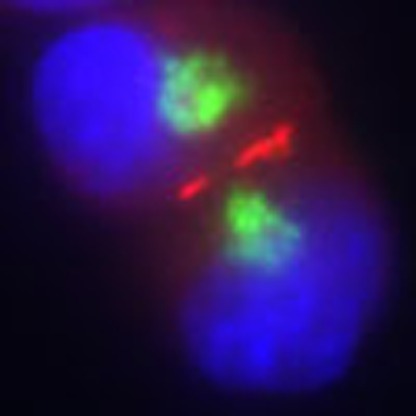

The formation of an apical lumen is a key step during epithelial tissue morphogenesis and function, and it is now well established that Rab-dependent endosome transport is responsible for driving individual cell polarity as well as *de novo* lumen formation^1^,^2^,^3^,^4^. Specifically, the Rab11 family of GTPases were shown to regulate the transport of vesicles carrying apical cargo to the site of the forming lumen, known as the apical membrane initiation site (AMIS)^1^,^2^,^5^,^6^,^7^,^8^. AMIS is a transient structure that contains many proteins, including the Par3/Par6 polarity complex, the Exocyst complex and tight junction (TJ) proteins such as ZO-1 and Cingulin (CGN)^1^,^2^,^5^,^7^,^8^. *De novo* formation of a single AMIS is an essential cellular step leading to the proper initiation and expansion of a single apical lumen^1^,^2^,^7^,^8^. Recent work from our and other laboratories has shown that midbody formation and midbody-dependent AMIS recruitment during telophase is the first symmetry-breaking event that determines the time and site of apical lumen formation^1^,^7^. However, the factors involved in AMIS recruitment to the midbody are still unknown and are the focus of this study.

In addition to midbody-dependent AMIS formation, apical endosome targeting and fusion at the AMIS is also an important step in apical lumen formation. Previous studies have begun to identify the mechanisms of apical endosome budding and targeting and have shown that apical endosome transport is governed by Rab11 GTPase bound to its effector protein known as Rab11 family interacting protein-5 (FIP5)^6^,^7^,^8^. The sequential interactions of Rab11/FIP5 targeting complex with Sorting Nexin-18 (SNX18) and Kinesin-2 regulate apical endosome formation and transport along central spindle microtubules during the initial steps of lumenogenesis^6^,^8^. Although it is known that these vesicles fuse with the plasma membrane at the AMIS, the specific mechanisms of targeting and tethering of Rab11/FIP5 endosomes to the AMIS are not fully understood. While several proteins, such as synaptotagmin-like proteins Slp2 and Slp4 as well as the Exocyst complex, were shown to be required for single-lumen formation^9^, it is unlikely that they alone can target endosome transport to the AMIS, since most of these factors localize and function at other subcellular locations in addition to the AMIS and/or midbody, thus limiting their ability to serve as AMIS-specific tethers for incoming apical vesicles.

Here, we investigate the machinery that mediates AMIS formation at the midbody, as well as the targeting/tethering of apical endosomes during lumenogenesis. We have identified CGN^10^ as a FIP5-binding protein and have shown that CGN serves as the tethering factor that ensures the fidelity of apical endosome targeting to the AMIS. We also show that CGN binds to the carboxy-terminal tails of midbody microtubules, and that this CGN and microtubule interaction may play a major role in recruiting the AMIS to the midbody during late telophase. Finally, we uncovered a novel and midbody-dependent role of Rac1-WAVE/Scar-induced actin polymerization during the initial steps of apical lumen formation. As the result of this data, we propose a new apical lumen formation model that explains how polarized endocytic membrane transport, midbody microtubules and branched actin cytoskeleton interact and function as a coincidence detection system that regulates the timing and fidelity of single apical lumen formation.

## Results

### CGN is a FIP5 binding protein concentrated at the AMIS

**Figure 1:**
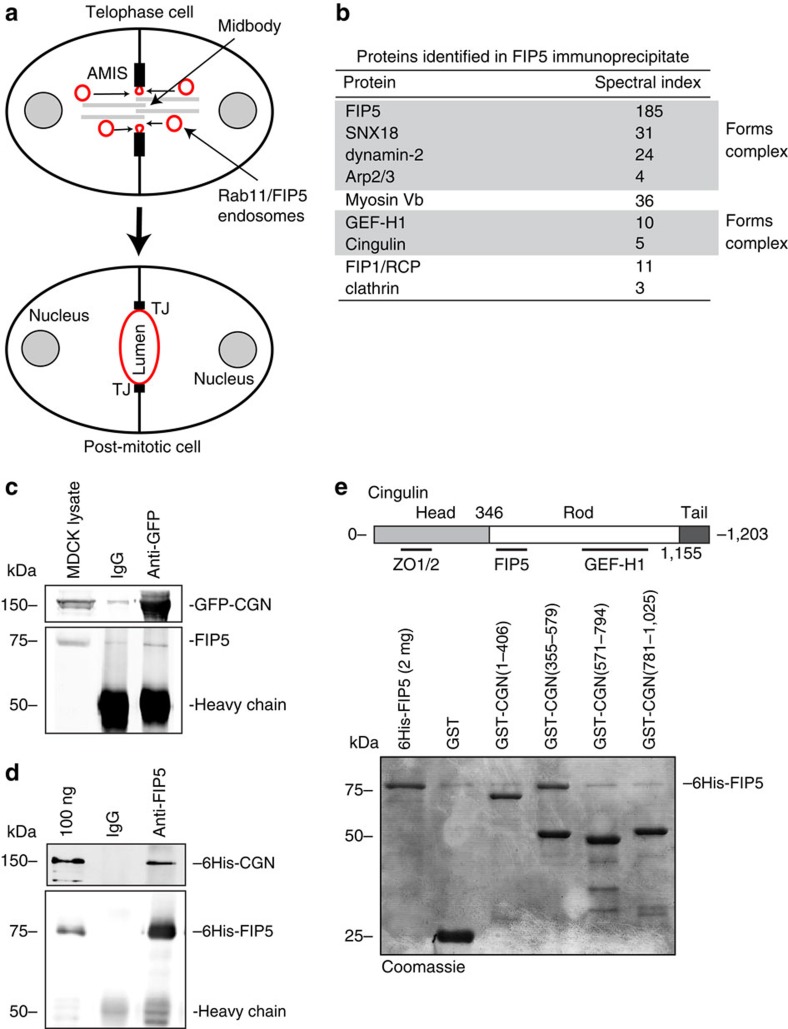
CGN is an AMIS-associated FIP5-binding protein. (**a**) Schematic model showing the formation of a nascent apical lumen. Rab11/FIP5 endosomes are transported along central spindle microtubules and fuse to the AMIS present at the midbody during late telophase. (**b**) List of proteins identified in FIP5 immunoprecipitate by mass spectroscopy analysis. Proteins known to be present in the same complex are highlighted together. (**c**) FIP5 co-immunoprecipitation with GFP-CGN from MDCK lysates. Images shown are the immunoblots after probing with anti-CGN (top gel) or anti-FIP5 (bottom gel) antibodies. (**d**) Co-immunoprecipitation of purified 6His-CGN with 6His-FIP5. Images shown are the immunoblots after probing with anti-CGN (top gel) or anti-FIP5 (bottom gel) antibodies. (**e**) Mapping 6His-FIP5 binding domain using glutathione bead pull-down assays. Image shown is Coomassie stained SDS–PAGE gel.

During *de novo* lumen formation the AMIS is established at the midbody during late telophase, marking the site of a future apical lumen^1^,^7^ (Fig. 1a). Following AMIS formation, Rab11/FIP5 apical endosomes are transported to the AMIS (Fig. 1a)^1^,^6^. What is not known are the mechanisms that target Rab11/FIP5 vesicles to the AMIS. To identify these targeting factors we immunoprecipitated FIP5 from polarized Madin–Darby canine kidney (MDCK) cells (Supplementary Fig. 1a). Many of the identified proteins (Fig. 1b) are already known to regulate apical vesicle transport, confirming the efficacy of the immunoprecipitation. In fact, SNX18, dynamin-2 and Arp2/3 are all known to form a complex that is essential for the budding of FIP5 apical endosomes^8^. Myosin Vb and FIP1 are other known components of apical endosomes^11^,^12^,^13^,^14^,^15^, and clathrin is a general vesicle coating factor^16^,^17^. Interestingly, we also identified CGN as a putative FIP5-binding protein (Fig. 1b, Supplementary Fig. 1a). CGN is a known AMIS marker that is concentrated almost exclusively around the midbody during late telophase^7^,^8^, thus making it a strong candidate to serve as a tethering factor mediating Rab11/FIP5 apical endosome targeting.

To further test whether CGN binds to FIP5 we immunoprecipitated GFP-CGN from MDCK cells stably expressing GFP-CGN. Consistent with our proteomics data, FIP5 co-immunoprecipitated with GFP-CGN (Fig. 1c). Since we have previously shown that FIP5-T276 phosphorylation inhibits FIP5 (ref. 7), we next co-immunoprecipitated CGN with either FIP5-T276A-GFP or FIP5-T276D-GFP and found that FIP5 phosphorylation mimetic FIP5-T276D did not bind to CGN (Supplementary Fig. 1b). Finally, purified recombinant 6His-FIP5 and 6His-CGN were also co-immunoprecipitated with anti-FIP5 antibody (Fig. 1d), further demonstrating that CGN directly binds to FIP5.

CGN contains three main domains: a large globular head, coiled-coil rod and short globular tail (Fig. 1e)^18^. The head domain is known to bind ZO1 and mediate CGN recruitment to TJs^18^,^19^ and the coiled-coil rod is required for CGN homo-dimerization and mediates binding to GEF-H1 (Fig. 1e)^18^,^20^. To map the FIP5-binding motif we used a GST pull-down assay and showed that FIP5 binds to the amino-terminal portion of the coiled-coil rod (amino acid region 355–579) (Fig. 1e). Since CGN (355–579) is located just after the large globular head domain, we refer to it as the CGN-coiled-coil-1 region (Fig. 1e).

### CGN is required for single apical lumen formation

**Figure 2:**
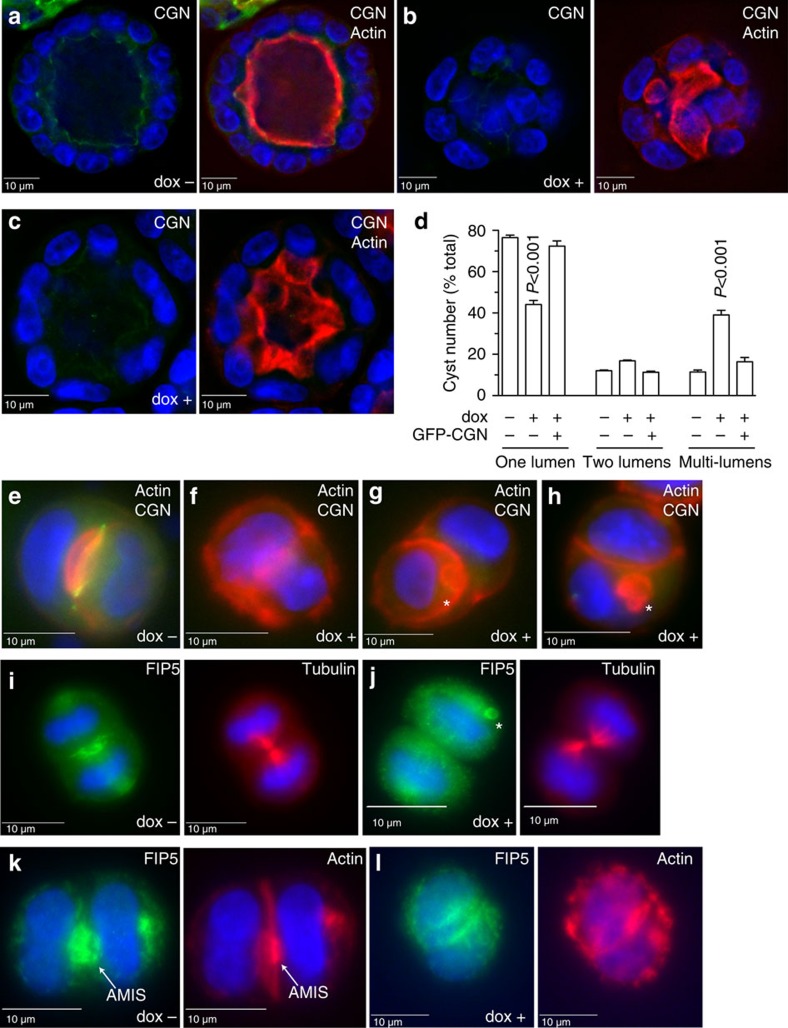
CGN is required for the formation of a single apical lumen. MDCK cells stably expressing CGN shRNA were embedded in 3D Matrigel and allowed to grow for 4 days (**a**–**d**) or 24 h (**e**–**l**) in the presence (dox+) or absence (dox−) of doxycycline. Cells were then fixed and stained with phalloidin-Alexa594 (**a**–**c**,**e**–**h**,**k**,**l**; red), anti-CGN (**a**–**c** and **e**–**h**; green), anti-tubulin (**i**,**j**; red) or anti-FIP5 (**i**,**j**; green) antibodies. Asterisks in (**g**,**h**,**j**) mark ectopic lumen formation sites. Arrows in (**k**) point to the AMIS. (**d**) shows quantification of cysts with one, two, or multiple lumens. Data shown are means and s.d. of three independent experiments. Consecutive images without an individual letter label show the same cell.

To assess the role of CGN in lumen formation, we created a doxycycline (dox) inducible MDCK-shCGN cell line (Supplementary Fig. 1c,d) and tested the effects of CGN knock-down (KD) on apical lumen formation. After embedding MDCK cells in Matrigel for 4 days, dox− cells produced single-lumen cysts (Fig. 2a,d) with CGN, FIP5, gp135 and actin outlining the apical domain (Fig. 2a, Supplementary Fig. 2a,c,i). In contrast, CGN knock-down led to increased formation of cysts with multiple lumens (Fig. 2b,d, Supplementary Fig. 2b,d,e,j). These multiple lumens are likely the result of apical cargo mistargeting, since CGN depletion did not have any effect on the angle and positioning of the mitotic spindle (Supplementary Fig. 3c–f). Furthermore, even the dox+ cells that contained a single lumen had defects in apical surface formation. As shown in Fig. 2c, Supplementary Fig. 2e, MDCK-shCGN cells appear to have a large apical plasma membrane surface that extends and folds into the luminal space. Significantly, the addition of shRNA-resistant GFP-CGN to dox+ cells rescued the single-lumen phenotype as well as the integrity of the apical surface (Fig. 2d, Supplementary Fig. 2f, *t*-test).

Our data suggest that CGN regulates formation of a single apical lumen by affecting targeting of Rab11/FIP5 endosomes. To test this we analysed MDCK-shCGN cells embedded in Matrigel for 12 h. Under these conditions, most embedded cells are either in late telophase, or just completed their first division and formed a nascent mini-lumen between two daughter cells^7^. To determine whether CGN knock-down affects Rab11/FIP5 endosome transport, we stained cells with anti-FIP5 antibodies. As previously reported, FIP5 and gp135 are predominantly concentrated at the AMIS (Fig. 2i,k, arrows; Supplementary Fig. 2g) as marked by either tubulin or actin. In contrast, in the dox+ treated cells FIP5 endosomes are distributed throughout the daughter cells, indicating that CGN is required for the targeting of FIP5 (Fig. 2j,l). Consistently, gp135, a well-established FIP5-endosome cargo protein, was also scattered throughout the cytosol (Supplementary Fig. 2h). Additionally, in some cells we could observe gp135 accumulating in enlarged cellular structures that may represent ectopic nascent lumens (Supplementary Fig. 2h, arrow).

Surprisingly, CGN knock-down also disrupted actin cytoskeleton localization (Fig. 2f–h,l). Typically, during lumenogenesis, actin clearly marks the surface of the apical lumen (Fig. 2e,k). In contrast, in dox+ treated cells actin accumulation at the apical plasma membrane is lost, and actin is instead present in distinct foci along the entire plasma membrane (Fig. 2f–h,l). In some cells we also observed large intracellular structures containing enriched actin (Fig. 2g,h, asterisks). These intracellular structures are likely either enlarged apical endosomes or ectopic apical lumen-like structures, since they also contain FIP5 (Fig. 2j, asterisk). CGN knock-down also changes the appearance of actin in two-dimensional cultures, where stress fibre formation is induced at the basal side of the epithelial monolayer (Supplementary Fig. 3a,b).

### The WAVE/Scar complex is present at the AMIS

**Figure 3:**
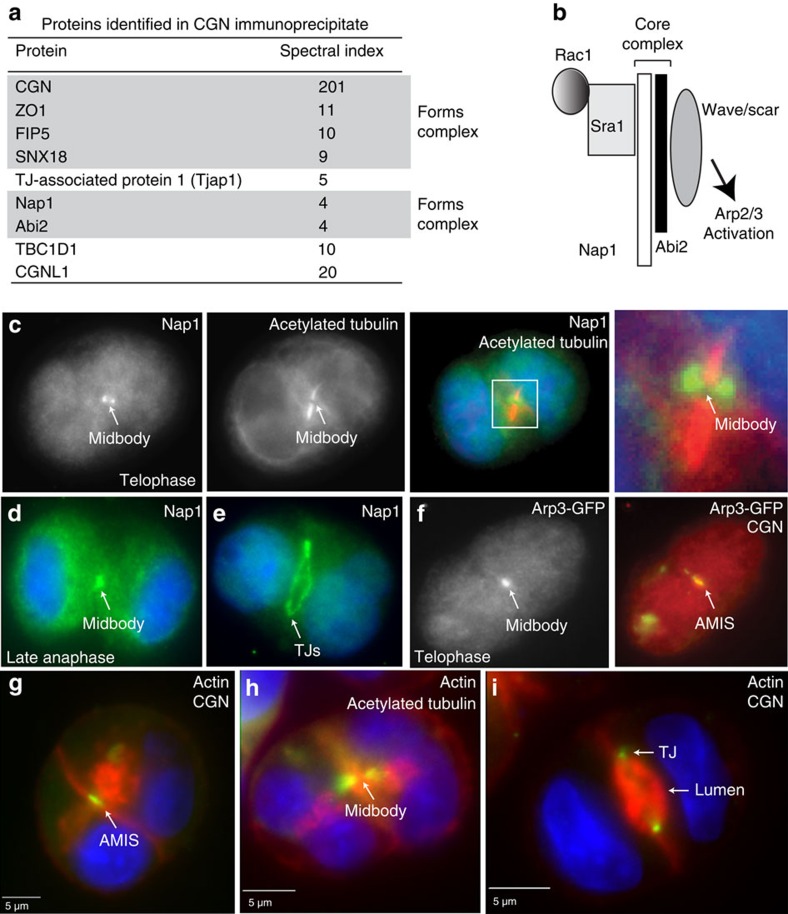
Components of the WAVE/Scar complex and branched actin filaments are present at the midbody during lumen formation. (**a**) List of proteins identified in CGN immunoprecipitate by mass spectroscopy. Proteins known to interact as a complex are highlighted together. (**b**) Schematic representation of the WAVE/Scar complex. (**c**–**i**) MDCK cells were embedded in 3D Matrigel and allowed to grow for 24 h. Cells were then fixed and stained with phalloidin-Alexa594 (**g**–**i**; red), anti-CGN (**f**,**g**,**i**), anti-acetylated tubulin (**c**; red and **h**; green) or anti-Nap1 (**c**–**e**; green) antibodies. In (**f**), cell is expressing Arp3-GFP. White arrows identify structures as labelled. Consecutive images without an individual letter label show the same cell.

Although we have previously shown that CGN and the AMIS are recruited to the midbody during late telophase^7^, the mechanisms mediating the establishment of the AMIS at the midbody remain unknown. Thus, we next synchronized MDCK cells at telophase and immuno-precipitated CGN. Isolated proteins were then analysed by mass spectroscopy and compared with those pulled down in an IgG control. Among the proteins identified only in anti-CGN (Fig. 3a) we identified ZO1, a known CGN-binding protein. Consistent with our finding that CGN is an FIP5-binding protein, we also identified FIP5 and SNX18 from anti-CGN antibody precipitates (Fig. 3a).

In addition to expected CGN-interacting proteins we also identified Nap1 and Abi2, two components of the WAVE/Scar complex (Fig. 3a,b). The WAVE/Scar complex is known to be activated by Rac1 and induces the polymerization of branched actin through the activation of Arp2/3 (Fig. 3b)^21^,^22^,^23^. To determine if the WAVE/Scar complex may play a role in the recruitment of CGN to the midbody, we first tested the localization of Nap1 and Arp3 during division of MDCK cells embedded in three-dimensional (3D) matrix. As shown in Fig. 3c, Nap1 co-localizes around the midbody during late telophase and also forms a ring structure localized to TJs after the formation of the nascent lumen (Fig. 3d,e), mirroring what we previously observed with CGN^7^. Likewise, Arp3 becomes concentrated at the midbody and co-localizes with CGN during AMIS formation (Fig. 3f). We also analysed the actin cytoskeleton during AMIS formation and lumen expansion, and detected an increase in actin polymerization around the midbody (Fig. 3g,h, Supplementary Fig. 4a). These actin structures emanate from the AMIS, suggesting that they could be actively playing a role in the establishment of the apical lumen. After formation of the nascent lumen, actin is still closely aligned with CGN, marking the apical surface and TJs (Fig. 3i). On the basis of these findings we hypothesized that the midbody-associated WAVE/Scar complex is helping to recruit the AMIS and may also drive apical lumen formation.

### Active Rac1 is required for AMIS formation at the midbody

**Figure 4:**
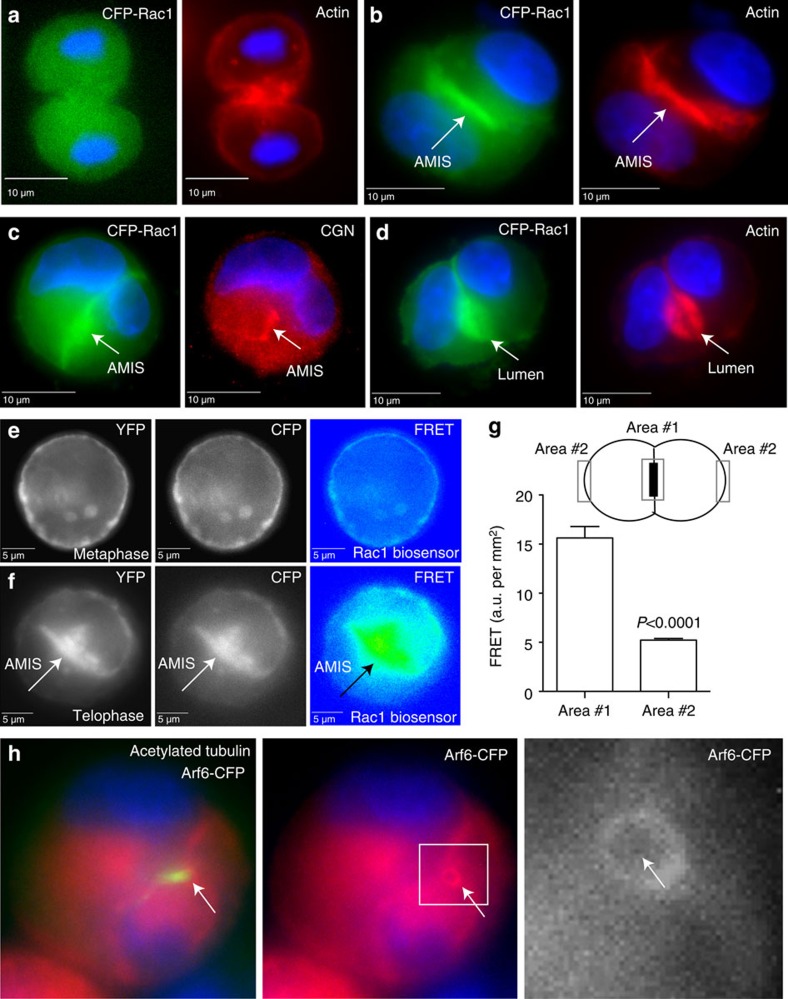
Rac1 is activated at the AMIS. (**a**–**d**) MDCK cells transiently expressing CFP-Rac1 (green) were embedded in 3D Matrigel and allowed to grow for 24 h. Cells were then fixed and stained with phalloidin-Alexa594 (**a**,**b**,**d**; red) or anti-CGN (c; red) antibodies. (**e**–**g**) MDCK cells transiently expressing FRET-based Rac1 biosensor were embedded in 3D Matrigel and imaged either at metaphase (**e**) or telophase (**f**). (**g**) shows quantification of FRET signal at the AMIS (area #1) and cell periphery (area #2) during telophase. Data shown are the means and s.d. derived from six cells. (**h**) MDCK cells transiently expressing CFP-Arf6 (red) were embedded in 3D Matrigel and allowed to grow for 24 h. Cells were then fixed and stained with anti-acetylated tubulin (red) antibodies. Arrows mark the midbody. Consecutive images without an individual letter label show the same cell.

The WAVE/Scar complex is activated by the binding of the small GTPase Rac1 (refs 21, 22, 23). Our finding that WAVE/Scar and therefore Rac1 may be involved in regulating AMIS formation during late telophase is somewhat unexpected, since it is well established that RhoA rather than Rac1 drives cleavage furrow formation and ingression during cytokinesis^24^,^25^,^26^. Importantly, it was demonstrated that RhoA is inactivated at late telophase to allow for the disassembly of the contractile actomyosin ring^27^. This suggests that during late telophase RhoA inactivation is followed by Rac1 activation, thus allowing a transition to Arp2/3-dependent branched actin cytoskeleton. To that end we analysed the localization of Rac1 during lumen formation (Fig. 4a–d). During anaphase, Rac1 is evenly distributed throughout the cytosol of dividing cells, consistent with Rac1 not being involved in the formation of the actomyosin ring (Fig. 4a). During late telophase, Rac1 becomes concentrated at the AMIS (Fig. 4b) where it co-localizes with filamentous actin and CGN (Fig. 4b,c). Upon formation of the nascent mini-lumen, Rac1 remains enriched at the apical plasma membrane (Fig. 4d), indicating its possible involvement in maintenance of apical polarity. While subcellular Rac1 localization can provide clues about its possible function, ultimately GTP-bound Rac1 is what activates WAVE/Scar and induces actin polymerization. To test the localization of active Rac1 we used an Rac1 biosensor^28^. As shown in Fig. 4e, in metaphase, activated Rac1 appears to be equally distributed along the entire plasma membrane. In contrast, once the cell enters telophase, Rac1 becomes activated at the AMIS (Fig. 4f,g).

Although these experiments directly link Rac1 activation to the establishment of the apical lumen, it does not explain the mechanism governing the recruitment and activation of Rac1 during late telophase. Previous studies have shown that Arf6 is recruited to the midbody during telophase^29^,^30^. It was suggested that Arf6 binds and recruits Tiam1, a known Rac1 GEF^31^. Consistent with this hypothesis, Arf6 can be observed at the midbody of the MDCK cells during lumenogenesis (Fig. 4h).

**Figure 5:**
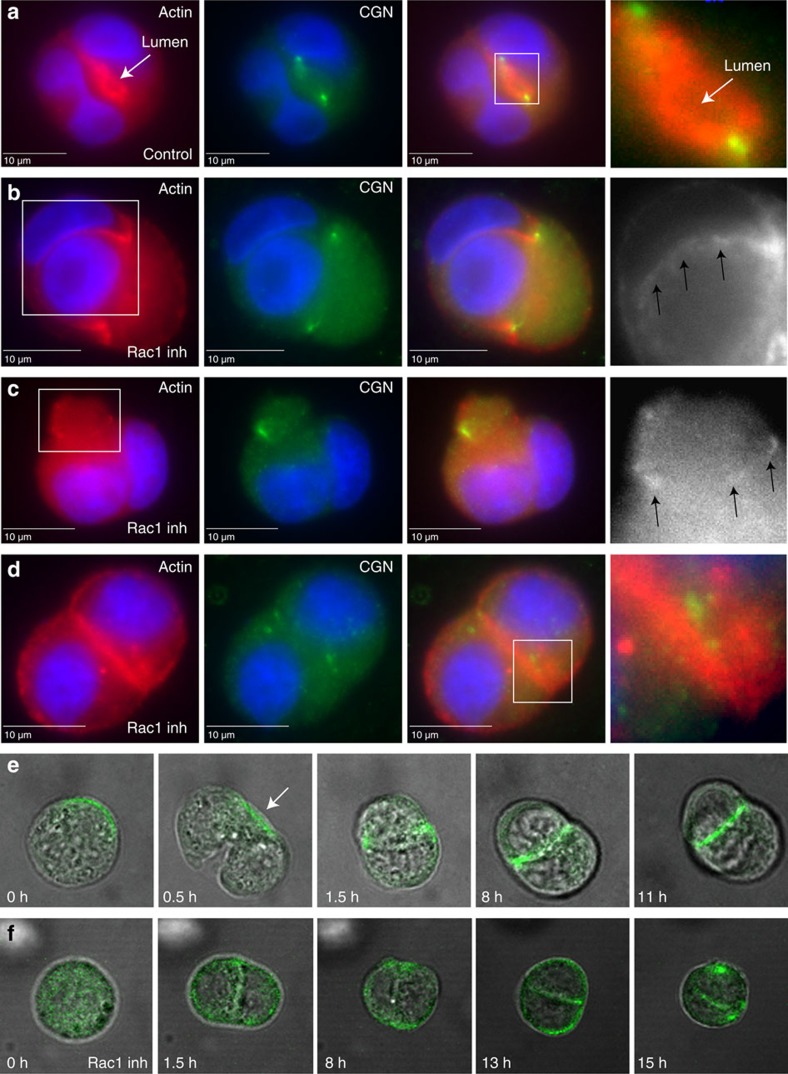
Rac1 inhibition affects midbody-associated AMIS formation. (**a**–**d**) MDCK cells were embedded in 3D Matrigel and allowed to grow for 24 h. Where indicated, cells were treated with 200 μM Rac1 inhibitor (**b**–**d**). Cells were then fixed and stained with phalloidin-Alexa594 (red) and anti-CGN (green) antibodies. White boxes indicate area zoomed in and shown in far right box. White arrows point to nascent lumen, black arrows point to areas of actin concentration. Consecutive images without an individual letter label show the same cell. (**e**,**f**) MDCK cells stably expressing GFP-CGN were embedded in 3D Matrigel. Untreated (**e**) or Rac1 inhibitor treated (**f**) cells were then imaged using time-lapse microscopy. In all cases, cell progression from metaphase to abscission was analysed. Arrow in (**e**) points to the midbody associated GFP-CGN.

We next wanted to determine if Rac1 plays a functional role in the establishment of the apical lumen. It has been previously shown that Rac1 knock-down leads to formation of inverted cysts with the apical pole of the cells facing the ECM, thus lacking a well-defined apical lumen^32^,^33^,^34^. However, the use of cell lines expressing Rac1 shRNA is difficult to interpret, since Rac1 is one of the main regulators of actin cytoskeleton. As a result, instead of shRNA, we used an inhibitor that specifically targets the Rac1 GEFs (Tiam1 and Trio). To ensure that we are inhibiting Rac1 only during the first mitotic division, we embedded MDCK cells into Matrigel for 4 h, followed by a 12-h incubation with Rac1 inhibitor. After 16 h, most of the cells successfully complete the first division, and the CGN-enriched TJ ring surrounds the nascent mini-lumen, which is always found between the two daughter cells (Fig. 5a). In contrast, cells treated with Rac1 inhibitor failed to form the CGN ring and apical lumen between the two daughter cells (Fig. 5b). Instead, CGN forms a TJ ring at the side of one of the daughter cells (Fig. 5b), despite the fact that cells successfully completed the first mitotic division (Fig. 5b; arrows mark the actin-rich plasma membrane between two nuclei). Occasionally we could also observe a small CGN and actin ring (Fig. 5c) that forms on the neck of a small bud-like structure on one of the daughter cells. Finally, in some cases the cells divide, but fail to polarize and form any TJs of nascent mini-lumens (Fig. 5d).

**Figure 6:**
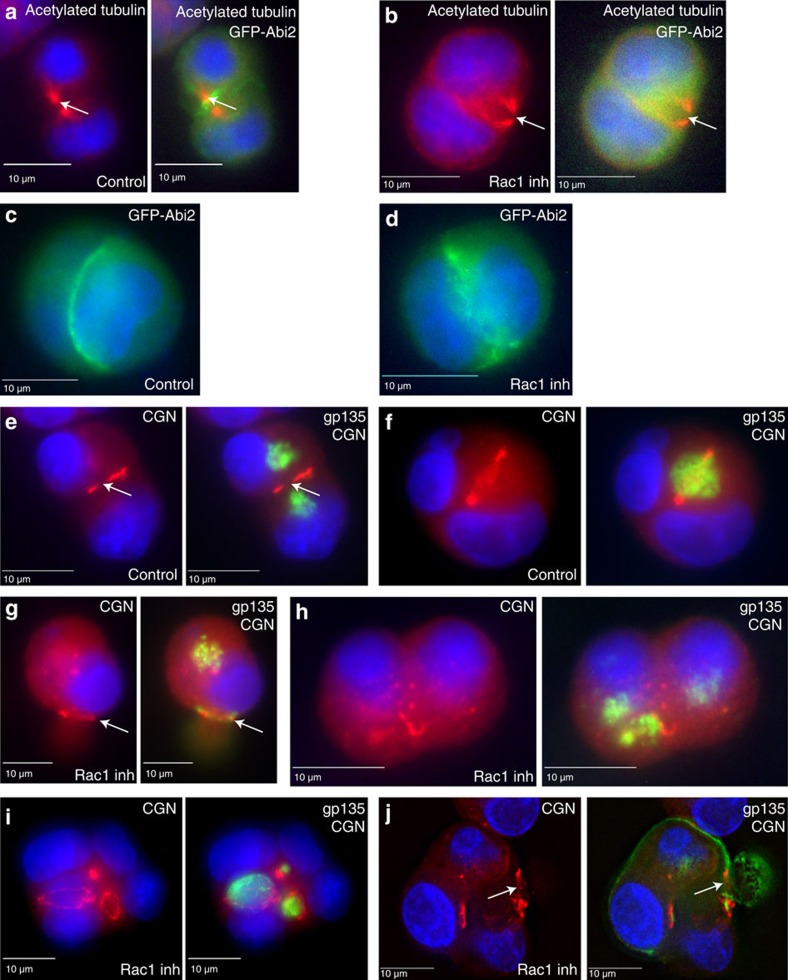
Rac1 is required for the gp135 targeting during apical lumen formation. (**a**–**d**) MDCK cells transiently expressing GFP-Abi2 were embedded in 3D Matrigel and allowed to grow for 24 h. Where indicated, cells were treated with 200 μM Rac1 inhibitor (**b**,**d**). Cells (**a**,**b**) were then fixed and stained with anti-acetylated tubulin (red) antibodies. White arrows (**a**,**b**) point to the midbody. (**e**–**h**) MDCK cells were embedded in 3D Matrigel and allowed to grow for 24 h. Where indicated, cells were treated with 200 μM Rac1 inhibitor (**g**,**h**). Cells were then fixed and stained with anti-gp135 (green) and anti-CGN (red) antibodies. White arrow in (**e**) points to the midbody. White arrow in (**g**) points to misplaced CGN. (**i**,**j**) MDCK cells were embedded in 3D Matrigel and allowed to grow for 2 days. Cells were treated with 200 μM Rac1 inhibitor for the first 12 h of the incubation. Cells were then fixed and stained with anti-gp135 (green) and anti-CGN (red) antibodies. White arrow in (**j**) points to misplaced CGN. Consecutive images without an individual letter label show the same cell.

To further analyse the role of Rac1 in regulating AMIS formation we followed GFP-CGN dynamics during MDCK cell division by time-lapse microscopy. Consistent with previously published work^7^, CGN accumulated at the midbody during telophase (Fig. 5e, arrow). Upon completion of cell division, CGN formed a ring between two daughter cells, an indication of apical lumen and TJ formation (Fig. 5e). In contrast, treatment of cells with Rac1 inhibitor blocked accumulation of CGN at the midbody and consequently prevented apical lumen formation and CGN accumulation at the TJs (Fig. 5f, Supplementary Fig. 5a). Since Rac1 activates the WAVE/Scar protein complex we next checked the localization of Abi2, the core subunit of the complex. Similar to Nap1, GFP-Abi2 also localized to the midbody during telophase (Fig. 6a), as well as to TJs after completion of the first division and the establishment of the nascent apical lumen (Fig. 6c). Incubation of dividing cells with Rac1 inhibitor blocked GFP-Abi2 recruitment to the midbody (Fig. 6b). Interestingly, even recruitment of GFP-Abi2 to the TJs was affected, although not completely inhibited (Fig. 6d). Taken together, these data indicate that Rac1 and WAVE/Scar-dependent formation of branched actin filaments at the midbody may also play a role in the correct placement of TJs around the midbody and nascent apical mini-lumen.

Since Rac1 is required for a recruitment of the AMIS to the midbody, Rac1 inhibition should lead to defects in targeting of apical proteins. To test this, we analysed localization of gp135, an apical plasma membrane protein delivered to the apical lumen via Rab11 and FIP5 endosomes. As previously reported^7^,^8^ in control cells gp135 is delivered to the midbody-associated AMIS (Fig. 6e). These gp135-containing endosomes eventually fuse to form nascent mini-lumen (Fig. 6f). Inhibition of Rac1 led to a failure of gp135 targeting to nascent lumen. Instead gp135-containing vesicles accumulated in multiple clusters within the cytosol (Fig. 6g). Significantly, in some cells we observe a CGN ring that forms at the neck of small bud-like structures, and gp135-containing vesicles could also be observed at those necks (Fig. 6g, arrows), confirming that CGN does function as a factor mediating apical endosome targeting/tethering. In some cases, after 16 h the cells underwent two rounds of division, thus generating small four-cell cysts. Typically, at this stage gp135 could already be observed enriched within small mini-lumen (Supplementary Fig. 2f). Rac1 inhibition led to the formation of multiple mini-lumens surrounded by CGN-containing TJs (Fig. 6i). However, ∼45% of all the cysts contained an inverted polarity, with the gp135-containing plasma membrane facing the Matrigel (Fig. 6j). These inverted cysts were similar to the ones observed in cells expressing dominant-negative Rac1 mutants^32^,^34^.

**Figure 7:**
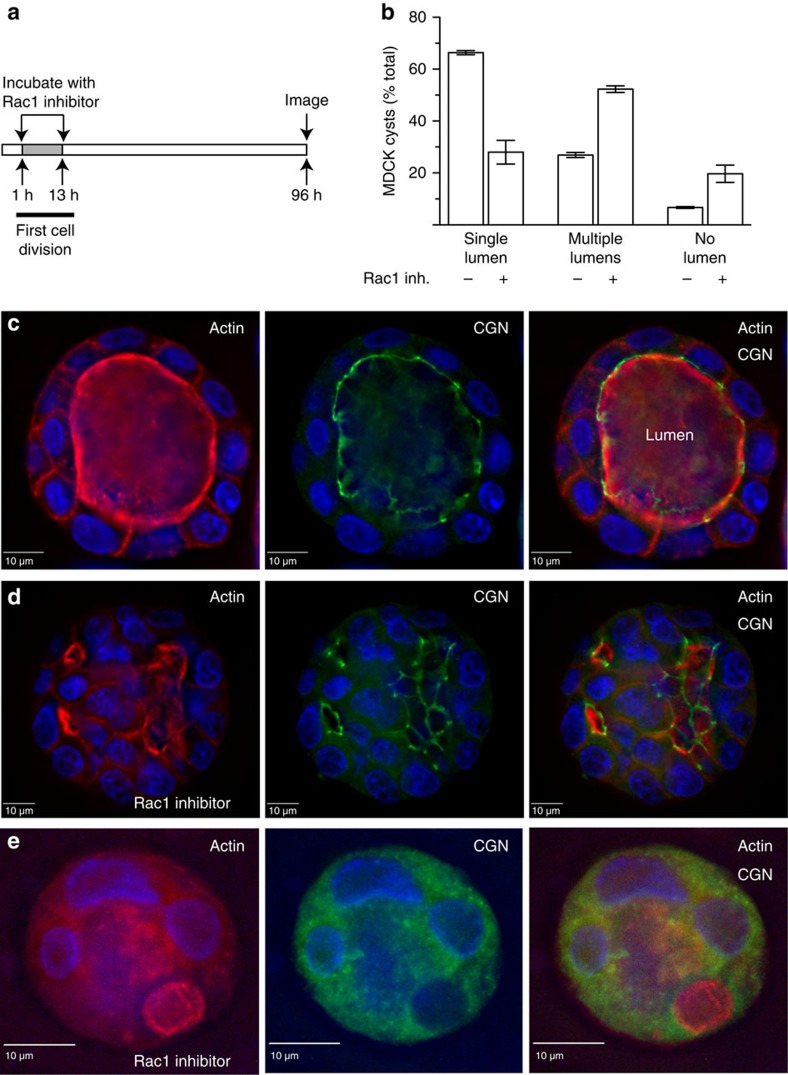
Rac1 is required for the formation of a single apical lumen. (**a**) Schematic representation showing timing of 12 h treatment with Rac1 inhibitor. (**b**–**e**) MDCK cells were embedded in 3D Matrigel and allowed to grow for 4 days in the absence (**c**) or presence (**d**,**e**) of Rac1 inhibitor. The timing of inhibitor addition is shown in (**a**). Cells were then fixed and stained with phalloidin-Alexa594 (red) and anti-CGN (green) antibodies. Consecutive images without an individual letter label show the same cell. (**b**) shows quantification of cells with single or multiple lumens. Data shown are the means and s.d. derived from three independent experiments.

If Rac1 is required for correct placement of the apical lumen and TJs, one would predict that inhibition of Rac1 during the first mitotic division should lead to the formation of mature cysts containing multiple lumens. To test this prediction, we treated embedded cells with Rac1 inhibitor for 12 h, then changed the media and allowed the cells to form mature cysts (4 days) (Fig. 7a). As reported previously, untreated cells developed into well-structured, single-lumen cysts (Fig. 7b,c) with CGN marking TJs and actin outlining the apical domain (Fig. 7c). The majority of cells treated with Rac1 inhibitor (Fig. 7b) formed cysts containing multiple lumens (Fig. 7d). It is likely that this multi-luminal phenotype is caused by incorrect placement of the AMIS (or multiple AMIS-like structures) since inhibition of Rac1 during the first cell division does affect mitotic spindle positioning and angle during subsequent cell divisions (Supplementary Fig. 3c–f). Surprisingly, after 4-day incubation we did not observe any inverted cysts, suggesting that re-activation of Rac1 (after inhibitor wash-out) was sufficient to correct polarity inversion but not the multi-luminal phenotype. In contrast, some cells did not form any apical lumen and had no clear CGN-rich TJs (Fig. 7b,e).

To further confirm that Rac1 pathway regulates apical lumen formation we treated cells with Arp2/3 inhibitor CK-666. To inhibit Arp2/3 only during the first cell division, we treated newly embedded cells with Arp2/3 inhibitor for 12 h, then changed the media and allowed the cells to form mature cysts (Supplementary Fig. 4b). Consistent with the involvement of Rac1 in mediating lumenogenesis, Arp2/3 inhibition led to the formation of cysts containing multiple lumens (Supplementary Fig. 4c–e). We again observed cysts with ectopic accumulations of CGN (Supplementary Fig. 4d,e, arrows).

To test whether Rac1 is required for apical lumen initiation only during the first mitotic division, we next treated MDCK cells with Rac1 inhibitor 24 h post embedding (Supplementary Fig. 5b). Surprisingly, treatment with Rac1 inhibitor after the initiation of a lumen during the first cell division still led to a multi-luminal defect (Supplementary Fig. 5c). However, these multi-luminal cysts were different from the ones formed by inhibiting Rac1. Typically, these cysts contained a single central primary lumen with one or more small mini-lumens located at the cyst periphery (Supplementary Fig. 5d). This indicates that even after the formation of a primary lumen Rac1 still plays a role in subsequent polarized cell divisions, and inhibiting Rac1 leads to the formation of multiple small secondary lumens. This is also consistent with previously published studies demonstrating that stable Rac1 knock-down leads to very dramatic effects on multiple stages of epithelia polarization^32^.

### CGN binding to tubulin mediates targeting to the midbody

**Figure 8:**
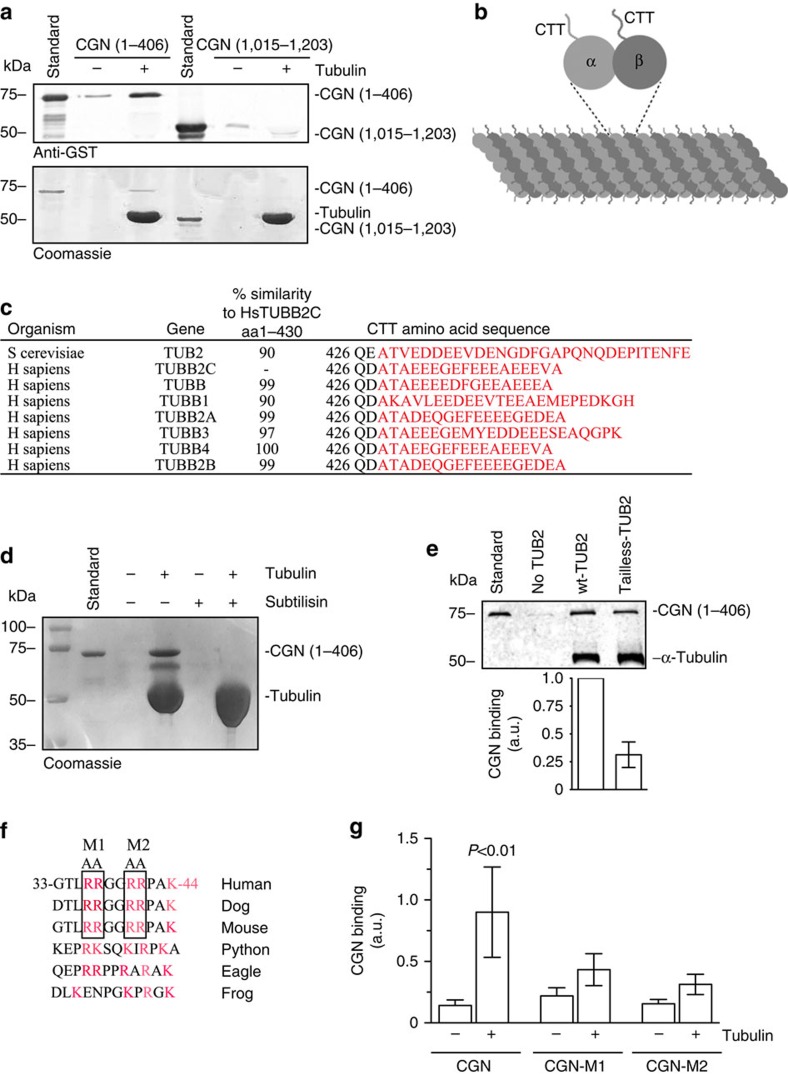
Electrostatic interactions mediate CGN binding to microtubule CTTs. (**a**) Microtubule binding assay showing amount of pelleted GST-CGN(1–406) and GST-CGN(1015–1203) when incubated with (+) and without (−) taxol-stabilized microtubules (tubulin). Anti-GST western blot (top gel), and Coomassie stained SDS–PAGE gel (bottom gel) are shown. (**b**) Diagram depicting positioning of α- and β-tubulin CTTs within polymerized microtubule. (**c**) Amino acid sequence alignment comparing CTTs of human and yeast β-tubulins. (**d**) Microtubule binding assay comparing amount of CGN(1–406) that pellets with wild-type tubulin and subtilisin-digested tubulin. Coomassie stained SDS–PAGE gel is shown. (**e**) Microtubule binding assay comparing CGN(1–406) pelleting with wild-type yeast tubulin and mutant yeast tubulin containing no β-CTT. Anti-tubulin and anti-CGN western blot is shown. (**f**) Amino acid sequence alignment comparing CGN basic patch in different vertebrate species. Boxes mark the sites mutated to alanine for binding analysis shown in (**g**). (**g**) Quantification comparing microtubule binding of wild-type CGN(1–406), CGN-M1 (R36/37A) and CGN-M2 (R40/41A). Data shown are the means and s.d. derived from four independent binding experiments.

While we have now shown that CGN is required for single-lumen formation, the mechanism ensuring the fidelity of CGN recruitment to the midbody is still unknown. The midbody is composed of a dense network of microtubules that have been shown to transport endosomes carrying apical cargo to the AMIS^6^,^7^. A recent study suggested that CGN can bind directly to the microtubules^35^, suggesting that microtubule binding may target CGN to the midbody. To test this possibility we first used an *in vitro* microtubule-binding assay to demonstrate that the CGN-head region (aa1–406), but not tail region (aa1015–1203), binds directly to polymerized microtubules (Fig. 8a).

To further analyse the mechanism of CGN and microtubule binding, we focused on the basic structure of microtubules. Microtubules are formed by polymerization of α-tubulin and β-tubulin heterodimers. Each α- and β-tubulin subunit contains an unstructured C-terminal tail (CTT) region that extends off the surface of polymerized microtubules (Fig. 8b). These CTTs contain a region rich in negatively charged amino-acid residues, which we refer to as the ‘acidic patch' (Fig. 8c). Tubulin CTTs are also susceptible to post-translational modifications, such as glutamylation^36^, which adds a chain of glutamate residues on a genetically encoded glutamate residue within the acidic patch and increases the negative charge of CTTs. While we are only beginning to understand the functional consequences of CTT glutamylation, midbody microtubules are also known to be highly glutamylated (Supplementary Fig. 6b)^37^. To determine whether CTTs may mediate CGN binding, we used subtilisin to cleave the CTTs from microtubules (Supplementary Fig. 6a) and compared CGN's ability to bind microtubules in the absence of CTTs (Fig. 8d). As shown in Fig. 8d, subtilisin treatment completely eliminated CGN binding to microtubules (Fig. 8d, lane 6).

We next tested whether CGN can bind to microtubules in which the CTTs are genetically ablated. Since mammalian cells have many β-tubulin isoforms, we decided to use yeast, as they contain only one gene for β-tubulin and can be engineered to express mutant β-tubulin that lacks the CTT region^38^. We first tested whether CGN can bind to microtubules made from purified wild-type yeast tubulins. As shown in Fig. 8e, despite differences in the primary sequences between human and yeast β-tubulin CTTs, human CGN can still bind to yeast microtubules. This further supports the putative involvement of the acidic patch in binding to CGN, since the presence of the acidic patch is preserved across species, despite sequence differences between human and yeast β-tubulin (Fig. 8c). We next tested the binding of CGN to mutant yeast tubulin (Supplementary Fig. 6c) and found that deletion of β-tubulin CTT leads to about 75% reduction in CGN binding (Fig. 8e), likely due to the loss of the CTT acidic patch.

Since CGN binds to CTT acidic patch we hypothesized that the CGN head domain should contain a basic patch. Indeed, CGN sequence analysis led to identification of an arginine and lysine-rich sequence that is present in all vertebrate sequences analysed (Fig. 8f). In mammals the core sequence of this basic patch consists of a highly conserved 36-RRGGRR-41 motif (Fig. 8f, boxes). To test whether this motif mediates microtubule binding we created two separate CGN mutants, Rs36/37As and Rs40/41As, and analysed mutant binding to microtubules (Fig. 8g). Consistent with the involvement of this basic patch, both mutants showed a significant decrease in CGN binding to microtubules (Fig. 8g, *t*-test). This result confirms that the binding is regulated by electrostatic interaction between the negatively charged tubulin tails and the basic patch of the CGN head region. The addition of charged modifications, such as glutamylation at the midbody, would enhance this interaction, and in our 3D embedded MDCK cells we observed both an enhancement in glutamylated tubulin around the midbody and focused localization around CGN at the AMIS (Supplementary Fig. 6b).

**Figure 9:**
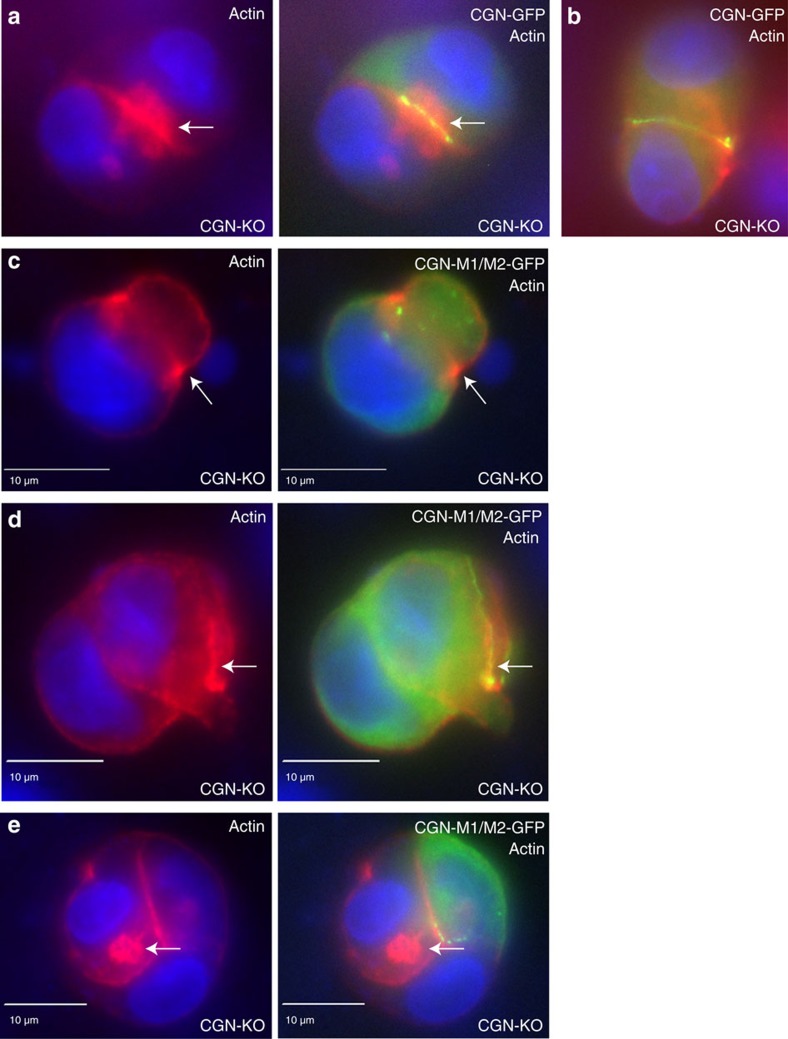
Mutation of basic patch disrupts subcellular CGN targeting. MDCK-CGN-KO cells were transfected with either wild-type CGN-GFP (**a**,**b**) or CGN-M1/M2-GFP mutant (R36/37/40/41A) (**c**–**e**). Cells were embedded in Matrigel, incubated for 12 h, fixed and stained with phalloidin-Alexa596. Arrows in (**a**) point to actin rich lumen surrounded by CGN-GFP-containing TJs. Arrows in (**c**) point to actin accumulation at the neck of bud-like structures. Arrows in (**d**) point to ectopic accumulation of actin and CGN rings. Finally, arrows in (**e**) point to intracellular formation of mini-lumens in CGN-KO cells.

We next mutated all four arginines to alanines (CGN-M1/M2-GFP) and tested the ability of these CGN mutants to localize at the lumen formation site. However, since CGN is known to homodimerize we were concerned that even in CGN shRNA-expressing cells, the remaining CGN may still target mutants to the appropriate location. To eliminate this possibility we used CRISPR/Cas9 to make an MDCK CGN knock-out cell line (MDCK-KO) (Supplementary Fig. 7a–c). Importantly, MDCK-KO recapitulated the multi-luminal phenotype observed in MDCK cells expressing CGN-shRNA (Supplementary Fig. 7d). We then used MDCK-KO cells to analyse the localization of CGN-M1/M2-GFP mutants during lumenogenesis. As shown in Fig. 9a,b, in the majority of cells (73.42±2.6% of cells) wild-type CGN-GFP localized to the TJs surrounding actin-rich single apical mini-lumens. In contrast, CGN-M1/M2-GFP-expressing cells largely failed to form a single apical lumen (26.58±2.8% of normal apical lumens; statistically different from wild-type CGN-GFP at *P*<0.0001; *t*-test), likely due to the fact that CGN-GFP mutants did not localize to the site of lumen formation (Fig. 9c–e). Interestingly, CGN-M1/M2-GFP localization was similar to CGN localization after treatment with Rac1 inhibitor. In some cells CGN-GFP mutants formed ectopic TJ-like structures that were also rich in actin (Fig. 9c,d), while in other cells CGN-GFP mutants were predominately cytosolic (Fig. 9e). Together, these data provide a targeted mechanism where cross-talk between actin and tubulin leads to the recruitment of CGN during establishment of the AMIS and formation of the apical lumen.

## Discussion

**Figure 10:**
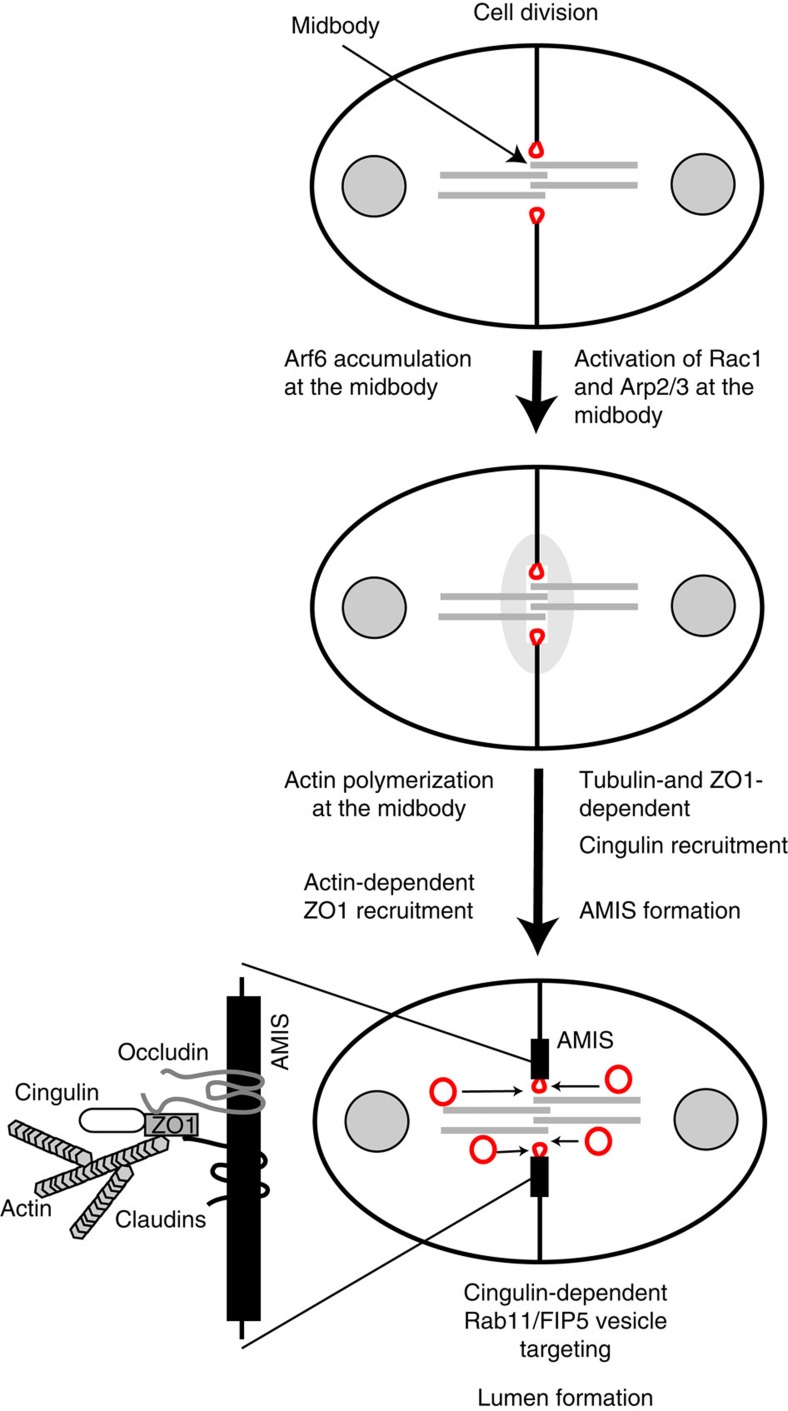
CGN interaction with midbody microtubules and Rac1-induced branched actin cytoskeleton is required for AMIS formation and apical lumen initiation. Model depicting pathways that lead to CGN recruitment and AMIS formation at the midbody as well as the targeting/tethering of Rab11/FIP5 apical endosomes to form a single apical lumen.

Recent work from many laboratories has demonstrated that *de novo* lumen formation relies on the targeted delivery of apical proteins to the AMIS, which forms around the midbody during cell division. It is now well established that these apical proteins are transported via Rab11/FIP5-containing apical endosomes, and that targeting of these endosomes to the AMIS is essential for the formation of a single apical lumen. However, while the mechanisms mediating apical vesicle targeting are beginning to emerge, two major questions remained unanswered: what factors are involved in establishing the AMIS at the midbody during the first cell division and how are apical Rab11/FIP5 endosomes specifically targeted to the AMIS. In this study we have shown that CGNbinds directly to FIP5 and functions as a targeting/tethering factor for Rab11/FIP5 endosomes during the early stages of lumen formation (Fig. 10). Our data support a model in which both the branched actin cytoskeleton and midbody microtubules play a role in initial establishment and maintenance of the AMIS. We have shown that the CTTs of microtubules bind to a basic patch located in the globular head region of CGN, and that this microtubule binding is likely to be required for initial CGN recruitment to the midbody (Fig. 10). We also demonstrate that Rac1 activation at the midbody leads to the recruitment of the WAVE/Scar complex and polymerization of branched actin filaments (Fig. 10). Importantly, polymerization of these branched actin filaments is also necessary for the formation of the AMIS at the midbody, and is also required for the formation and expansion of a single apical lumen (Fig. 10). How Rac1 is activated at the midbody during late telophase remains unclear, but it is possible that Arf6 mediates localization of the Rac1 GEF Tiam1 at the midbody. Indeed, Arf6 was shown to be enriched at the midbody^30^ and was also shown to be required for apical lumen formation^39^,^40^.

While Arf6 likely plays a role in activating Rac1 at the midbody-associated AMIS, there are also other pathways that can regulate actin dynamics during lumenogenesis. CGN itself recently emerged as a key hub that regulates the actin cytoskeleton. It was shown that CGN directly binds to GEF-H1, a GEF for RhoA^41^. Additionally, CGN was also shown to interact with MgcRacGAP^42^, although it remains controversial whether MgcRacGAP is a GAP for RhoA or Rac1 (refs 42, 43, 44). Finally, CGNL1 is known to bind to CGN as well as Tiam1, thus providing another pathway of activating Rac1 at the AMIS^41^. Consistent with these observations, CGN, RhoA and Rac1 were shown to be important for TJ stability in polarized epithelial cells^42^,^43^.

In addition to regulating the localization of the AMIS, midbody-associated branched actin is also likely involved in mediating apical endosome targeting. Myosin-Vb is well known to be required for apical lumen formation and is present at the Rab11/FIP5 endosomes^13^,^14^,^15^. Consistent with that, we also isolated Myosin-Vb as a protein that is associated with Rab11/FIP5 protein complex (Supplementary Fig. 1a). It has been proposed that Myosin-Va, a closely-related Myosin-Vb isoform, functions as a tether, sequestering melanosomes at the actin-rich cellular protrusions^45^. Thus, it is likely that midbody-associated actin flares may also help to sequester Myosin-Vb containing Rab11/FIP5 endosomes at the midbody and AMIS.

Taken together, we propose a new model of how the AMIS is recruited and maintained at the midbody during cell division (Fig. 10). The requirement of both branched actin and glutamylated microtubules serves as a coincidence detection system that allows for the establishment of a single apical lumen. Similarly, targeting of apical endosomes to the AMIS also depends on several factors. It was previously demonstrated that the Exocyst complex and Slps are required for the formation of a single apical lumen^46^,^47^,^48^. Here we show that CGN and branched actin are also required for Rab11/FIP5 targeting. Thus, it is becoming clear that lumen formation is dependent on a combination of overlapping pathways that occur at the right place and the right time to mediate lumen formation. This ever-growing complexity of apical lumen formation pathways may also be needed to allow for tissue-specific differences in the dynamics and timing of apical lumen formation. Indeed, mammary epithelial tissues appear to rely more on apoptosis-dependent lumen formation (known as cavitation)^49^. Consistent with that, recent work has shown that Rac1 may not play a key role in lumen formation during mammary tissue morphogenesis *in vivo*^50^.

Although this study has greatly expanded our knowledge of lumen formation, there are still many questions left to explore. Most of the *de novo* lumen formation studies have been conducted using *in vitro* models; thus it remains to be understood how apical endosome targeting and AMIS formation machinery function within the context of much more complex *in vivo* models. Additionally, during tissue morphogenesis, apical lumen formation relies not only on initiation of nascent mini-lumen, but also on a coalescence of these mini-lumens to form a final functioning apical space. While lumen coalescence clearly involves extensive remodelling of apical and basolateral plasma membrane, the mechanisms governing coalescence remain largely unclear. Thus, future studies will be needed to dissect the roles of CGN and FIP5 during the formation and coalescence of a single apical lumen *in vivo*.

## Methods

### Plasmids and antibodies

Rabbit polyclonal anti-CGN antibodies were prepared^8^ using recombinant purified human CGN head (aa1–406) and tail (aa1015–1203) fragments (Proteintech, Chicago, IL). Antibodies were affinity purified using recombinant CGN head and tail fragments conjugated to Affi-Gel 10 resin (Bio-Rad Laboratories, Hercules, CA) and eluted with 0.1 M glycine buffer, pH 2.5. Previously made rabbit polyclonal anti-FIP5 antibodies were also used^8^,^51^. Monoclonal mouse anti-acetylated tubulin antibodies were purchased from Sigma-Aldrich (St Louis, MO). Rabbit anti-Nap1 antibodies were purchased from NOVUS Biologicals (Littleton, CO). Mouse monoclonal anti-glutamylated tubulin antibodies were purchased from AdipoGen Life Sciences (San Diego, CA). AlexaFluor-594- and AlexaFluor-488-conjugated anti-rabbit and anti-mouse secondary antibodies were purchased from Jackson ImmunoResearch Laboratories (West Grove, PA). AlexaFluor-568-phalloidin was purchased from Life Technologies (Carlsbad, CA). The IRDye 680RD Donkey anti-mouse and IRDye 800CW donkey anti-rabbit secondary antibodies used for western blotting were purchased from Li-Cor (Lincoln, NB).

The GFP-Nap1 and Arp3-GFP were received from the lab of Giorgio Scita (University of Milan)^52^. GFP-CGN was received from the lab of Sandra Citi (University of Geneva)^53^. The Rac1-biosensor was received from the lab of Michiyuki Matsuda (Kyoto University)^28^. CFP-Rac1 was created by cloning Rac1 cDNA into a pECFP-C1 vector (Clontech, Palo Alto, CA). Antibody concentrations ranged from 0.1 to 0.5 mg ml^−1^, and were used at a 1:100 dilution.

### CGN knock-out by CRISPR/Cas9 in MDCK cells

The guide sequence targeted to exon 2 of canine CGN (Supplementary Fig. 7a) was cloned in lentiCRISPR vector that also contains Cas9 ORF^54^. MDCK cells were then transfected and cells expressing Cas9 and CGN guide were selected using puromycin. Multiple MDCK clonal cell lines were isolated and tested for the absence of CGN using western blotting. A cell line that did not contain any detectable endogenous CGN (Supplementary Fig. 7b,c) was then chosen for further analysis. This cell line was then genotyped by amplifying and sequencing the CRISPR target region and was found to have mutations in both copies of CGN leading to a premature STOP codon (Supplementary Fig. 7a).

### Immunoprecipitation and proteomic analysis

Immunoprecipitation of FIP5 and CGN was performed using MDCK cell lysates^8^. Cell lysates were harvested in phosphate-buffered saline (PBS) containing 1% Triton X-100 and phenylmethylsulfonyl fluoride (PMSF). Lysates were then incubated overnight at 4 °C with anti-FIP5 antibody, anti-CGN antibody or non-specific rabbit IgG control conjugated to protein A-sepharose beads (Sigma-Aldrich, St. Louis, MO). The beads were then pelleted and washed. To identify interacting proteins, immunoprecipitates were eluted with 1% SDS and separated on 7–15% SDS/PAGE gradient gel. Distinct bands present only in anti-FIP5 immunoprecipitate but not IgG control were cut out and analysed by mass spectroscopy^55^. Briefly, gel pieces were destained in 200 μl of 25 mM ammonium bicarbonate in 50% acetonitrile for 15 min and washed twice with 200 μl of 50% acetonitrile. Disulfide bonds in proteins were reduced by incubation in 10 mM dithiothreitol at 60 °C for 30 min; cysteine residues were alkylated with 20 mM iodoacetamide in the dark at room temperature for 45 min. After alkylation, 100 ng of trypsin was added to each sample and allowed to rehydrate the gel plugs at 4 °C for 45 min and incubated at 37 °C overnight. Samples were measured on a Velos Orbitrap mass spectrometer (Thermo Fisher Scientific) coupled to an Eksigent nanoLC-two-dimensional system through a nanoelectrospray LC−MS interface. Peptides were separated on a house-made 100 μm inner diameter × 150 mm fused silica capillary packed with Jupiter C18 resin (Phenomex). Data acquisition was performed by using the instrument supplied Xcalibur (version 2.0.6) software. The mass spectrometer was operated in the positive ion mode; the peptide ion masses were measured in the Orbitrap mass analyser, whereas the peptide fragmentation was performed by using either higher-energy collisional dissociation or electron transfer dissociation in the linear ion trap analyser by using default settings. Ten most intense ions were selected for fragmentation in each scan cycle; fragmented masses were excluded from further sequencing for 90 s. MS/MS spectra were extracted from raw data files and converted into Mascot generic format (MGF) files by using a PAVA script (University of California, San Francisco). These mgf files were then independently searched using an in-house Mascot server (Version 2.2.06, Matrix Science). Mass tolerances were ±15 p.p.m. for MS peaks and ±0.6 Da for MS/MS fragment ions. For all higher-energy collisional dissociation spectra, fragment ion tolerances were set to 0.05 Da. Trypsin specificity was used, allowing for one missed cleavage. Met oxidation, protein N-terminal acetylation and peptide N-terminal pyroglutamic acid formation were allowed for variable modifications while carbamidomethyl of Cys was set as a fixed modification. To identify CGN-binding proteins, immunoprecipitates were eluted from the beads with 0.1 M glycine pH 2.0 and prepped for mass spectroscopy in a similar manner, without the gel extraction steps and starting with the addition of trypsin.

### Protein expression and purification

Full-length 6His-FIP5 and 6His-CGN were produced in baculovirus using the transfer vector pVL1392 (ref. 8). In brief, 106 Sf9 cells were seeded into a six-well plate, and the Bacfectin–DNA mixture was added dropwise. After 5 days, the P1 viral stock was harvested and further amplified to P2 and P3 stages. For protein production, 1 l of Sf9 cells at 2 million cells per ml was infected with 2 ml of P3 viral stock (approximate MOI of 0.5) and harvested after 65 h. Cells were lysed in 50 mM Tris buffer, pH 7.5, containing 300 mM NaCl, and the cleared lysate was loaded onto a Ni-NTA column. Eluted 6His-FIP5 or 6His-CGN was dialysed overnight against buffer (50 mM Tris, pH 7.5, 300 mM NaCl and 5 mM BME) and frozen in liquid nitrogen. Yields were typically 3–5 mg l^−1^ with an estimated purity of >75%. GST-CGNaa1-406, GST-CGNaa355-579, GST-CGNaa571-794 and GST-CGNaa781-1025 fragments were expressed using the pGEX-4T plasmid (provided by Sandra Citi^18^) and purified using the BL21-(FE3) RIPL *Escherichia coli* strain^8^. Briefly, *E. coli* were lysed using a French press and then incubated with glutathione agarose beads (Sigma-Aldrich). Beads were then washed with PBS and the GST-protein was eluted with 25 mM glutathione (GE Healthcare). Final protein concentrations were determined using Bradford protein assay (Bio-Rad Laboratories).

### Glutathione bead pull-down assays

GST pull-down assays were performed^8^. Glutathione beads (50 μl) were coated with 10 μg GST fusion protein or GST control in PBS and incubated with specified amounts of soluble recombinant protein or MDCK cell lysates (PBS, 1% Triton X-100) in a final volume of 0.5 ml of reaction buffer (50 mM HEPES, pH 7.4, 150 mM NaCl, 5 mM MgCl_2_, 0.1% Triton X-100, 0.1% bovine serum albumin (BSA) and 1 mM PMSF). Samples were incubated at 25 °C for 1 h while rotating, pelleted by centrifugation at 2,000*g* for 3 min, rinsed with reaction buffer (1 ml × 3), and bound proteins were eluted with 1% SDS, analysed by SDS–PAGE, and stained with Coomassie blue or immunoblotted and scanned using a Li-Core Odyssey scanner.

### Cell culture

Parental MDCK-II cells (ATCC, Manassas, VA) were tested for mycoplasma, cultured in DMEM with glucose, L-glutamine, 10% FBS, and supplemented with penicillin and streptomycin. In two-dimensional cultures, MDCK cells were grown on collagen-coated Transwell filters for 4 days. 3D cultures of MDCK cells^8^ actively dividing were mixed in media to create a 25% Matrigel solution that was plated on eight-well slides (about 12 μl per well). The Matrigel–cell mixture was allowed to harden for 30 min at 37 °C, and 400 μl of medium was added. The cells were incubated for the indicated period of time and the media was changed every other day. 3D cell cultures were stained according to a modified previously published protocol^49^. In brief, 3D cultures were fixed with 3% paraformaldehyde for 20 min, permeabilized with PBS and 0.5% Triton X-100 for 10 min, and quenched three times for 15 min each wash with a glycine/PBS solution (130 mM NaCl, 7 mM Na_2_HPO_4_, 3.5 mM NaH_2_PO_4_ and 100 mM glycine). Cells were incubated in primary block (10% FBS, 130 mM NaCl, 7 mM Na_2_HPO_4_, 3.5 mM NaH_2_PO_4_, 7.7 mM NaN_3_, 0.1% BSA, 0.2% Triton X-100 and 0.05% Tween-20) for 4 h, followed by incubation in secondary block (primary block with 20 μg ml^−1^ goat anti-mouse F(ab′)_2_ fragments) for 1 h. After washing, cells were left overnight in primary block with primary antibody and Hoerscht nuclear stain. Cells were then washed and incubated for 1 h with secondary antibody in primary block. Cells were washed, dried for 1 h and mounted with VectaShield.

### Microscopy

All fixed cells were imaged with an inverted Axiovert 200M microscope (Carl Zeiss) with a × 63 oil immersion lens and QE charge-coupled device camera (Sensicam). Z-stack images were taken at a step size of 100–500 nm. Image processing was performed using 3D rendering and exploration software Slidebook 5.0 (Intelligent Imaging Innovations).

For live imaging, about 50,000 GFP-CGN MDCK cells were embedded in a 25% Matrigel solution and plated on a glass bottom dish, and 200 μM Rac1 inhibitor was added to treated samples. For imaging, the dishes were placed in a heat- and humidity-controlled chamber (37 °C, 5% CO_2_/95% air) on the stage of an inverted Zeiss LSM510 Meta confocal microscope. Cells were brought in focus using a × 10 objective, and z-stack images (2 μm steps) were acquired every 30 min for a 12–24 h period using a × 63 oil objective.

For fluorescence resonance energy transfer (FRET) analysis, cells were transfected with Raichu-Rac1 biosensor and embedded into 3D Matrigel matrix. After 24 h, cells were then imaged and corrected FRET (cFRET) was calculated using Intelligent Imaging Innovation three-dimensional rendering and exploration software^56^ with the equation cFRET=FRET−0.4 × CFP−0.037 × YFP.

### MDCK cell lines

Tet-inducible shRNA cell lines were created^8^ using canine CGN-shRNA sequences (5′- GATCCCCAGAGCATGTTCCAGAAGAATTCAAGAGATCTTTCTGGAACATGCTCTTTTTTA -3′) cloned into the pHUSH retroviral expression vector, transfected into MDCK cells using Lipofectamine 2000 (Invitrogen), and grown in media supplemented with tet-free FBS (Takara Bio) and 1 μg ml^−1^ of puromycin. Selected colonies were then grown in the presence of 1 μg ml^−1^ of dox for 72 h and tested for knock-down by western blotting. The GFP-CGN MDCK cell line was generated as previously described^57^.

### Rac1 inhibition studies

The Rac1 inhibitor (NSC 23766) was purchased from Santa Cruz Biotechnology. Cells embedded in 3D Matrigel were allowed to adjust to media for 2 h and treated with 200 μM inhibitor. For 4-day cysts, the inhibitor was removed after 12 h treatment by replacing media.

### Yeast tubulin purification

*Saccharomyces cerevisiae* (budding yeast) strains yJM0596/YEF473A MATa ura3-52 lys2-801 leu2-Δ1 his3-Δ200 trp1-Δ63 (wild type) and yJM0282 MATa tub2-430Δ+331::TRP1 ura3-52 lys2-801 leu2-Δ1 his3-Δ200 trp1-Δ63 (Beta tailless)^38^ were used as a tubulin source. Single-colony isolates of each strain were selected and cultured individually in 5 ml of rich yeast extract-peptone-dextrose (YPD) media overnight at 30 °C. Three hundred microlitres of the saturated overnight culture was used to inoculate 10 l of rich media (YPD) and grown to log phase at 30 °C ∼24 h after inoculation. The culture was collected by centrifugation and pelleted (3,500 r.p.m., 15 min at 4 °C, J-6B; Beckman Coulter, Brea, CA). This yielded between 120 and 140 g of wet cell pellet. Pellets were frozen at −80 °C until ready to purify. Purification was performed^58^ with the addition of an anion exchange chromatography step. After the budding yeast tubulin was eluted off the TOG column it was dialysed overnight in 2 l of BRB80 pH 6.9, 50 μM GTP. After dialysing the purified budding yeast tubulin, anion exchange chromatography was preformed using HiTrap Q HP (1 ml=1 column volume (CV); GE Healthcare, Buckinghamshire, UK) pre-equilibrated in BRB80 pH 6.8 (wash buffer) at 1 CV per min. The tubulin was eluted using 1 M NaCl in wash buffer. Peak fractions were determined by Bradford assay and pooled and dialysed in BRB80, 50 μM GTP pH 6.8. The concentration of the tubulin was determined by measuring the absorbance at 280 nm using a NanoDrop spectrophotometer (NanoDrop Technologies, Wilmington, DE) and the calculated extinction coefficient and molecular weight. Glycerol was added to 10% before the tubulin was aliquoted, snap frozen in liquid nitrogen, and stored at −80 °C.

### Microtubule binding assays

The microtubule binding assay was performed according to Microtubule Binding Protein Spin-down Assay Kit (Cytoskeleton). Briefly, 20 μl of taxol-stabilized microtubules (0.5 mg ml^−1^) was incubated with 2 μg test protein and 40 μg BSA in 200 μl general tubulin buffer (80 mM PIPES pH 7.0, 2 mM MgCl_2_ and 0.5 mM ethylene glycol tetraacetic acid (EGTA) for 30 min. The solution was placed on top of a 100 μl cushion buffer (80 mM PIPES pH 7.0, 1 mM MgCl_2_, 1 mM EGTA and 60% glycerol), spun down at 100,000*g*, supernatant removed, and the pellet resuspended in 1 × loading buffer. The binding was analysed by SDS–PAGE and stained with Coomassie blue or transferred for western blotting. For yeast tubulin binding, the microtubules were polymerized at 30 °C in a water bath and the rest of the experiment carried out at the same temperature in the same manner.

### Western blots

Some western blots are shown in their entirety in the figures. Whenever modified, all uncropped western blots can be found in Supplementary Fig. 8.

### Data availability

The authors declare that all data supporting the findings of this study are available within the article and its Supplementary Information files or from the corresponding author upon reasonable request.

## Additional information

**How to cite this article:** Mangan, A. J. *et al*. Cingulin and actin mediate midbody-dependent apical lumen formation during polarization of epithelial cells. *Nat. Commun.* 7:12426 doi: 10.1038/ncomms12426 (2016).

## Supplementary Material

Supplementary Information Supplementary Figures 1-8
