# Supplementary material for: Cingulin and actin mediate midbody-dependent apical lumen formation during polarization of epithelial cells
Source: Nat Commun. 2016 Aug 3;7:12426. doi: 10.1038/ncomms12426 (PMC4976216; doi:10.1038/ncomms12426)
Supplement: Supplementary Information — Supplementary Figures 1-8 [file ncomms12426-s1.pdf]

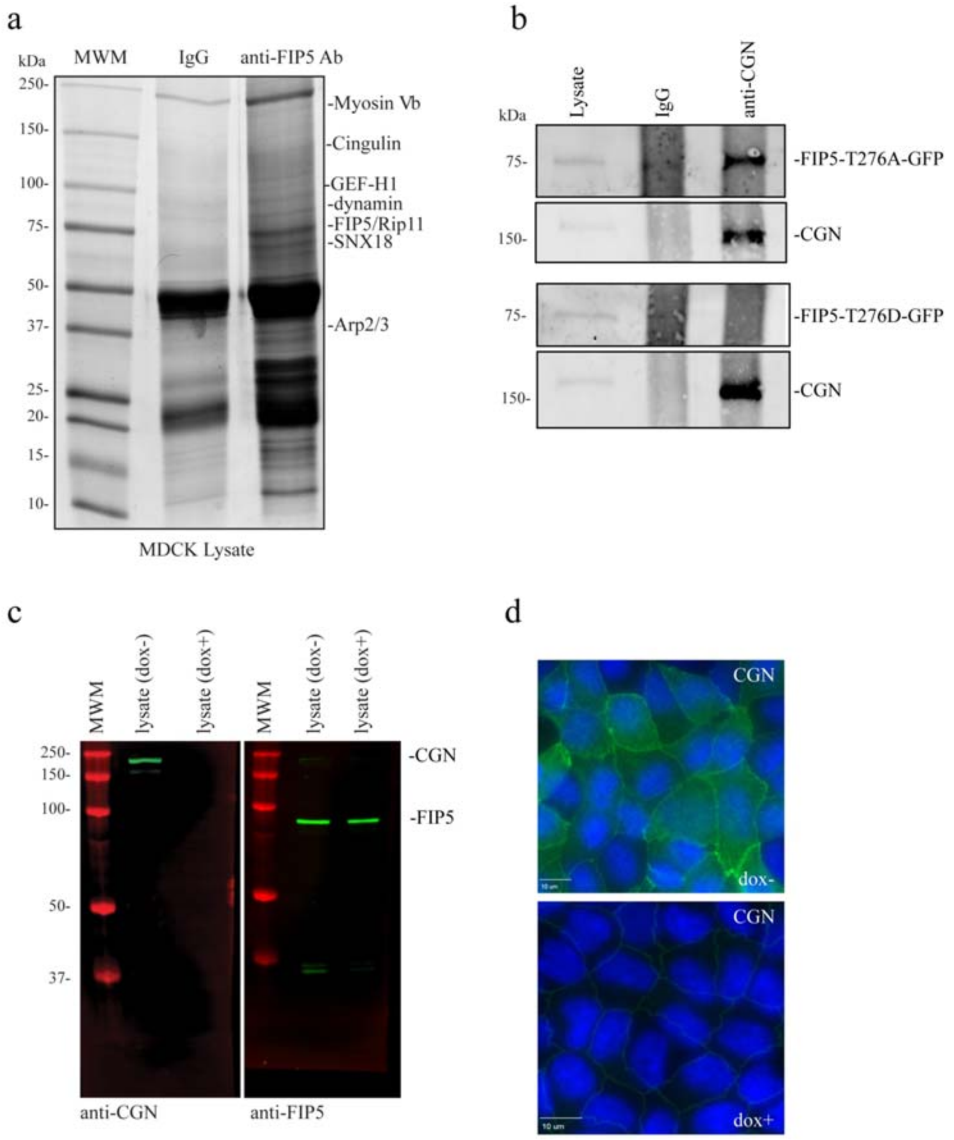

Supplementary Figure 1. CGN is a FIP5-interacting protein

(a) Coomassie-stained SDS-PAGE gel of FIP5 immunoprecipitates.

(b) Comparison of GFP-FIP5 mutant co-immunoprecipitation with anti-CGN antibody from MDCK cells transiently expressing either FIP5-T276A-GFP or FIP5-T276D-GFP.

(c) MDCK cells stably expressing tet-inducible CGN shRNA were grown in the presence or absence of doxycycline for 4 days. Cell lysates were then analyzed by immunoblotting with anti-CGN (left gel) and anti-FIP5 (right gel) antibodies.

(d) MDCK cells stably expressing tet-inducible CGN shRNA were grown on Transwell filters in the presence or absence of doxycycline for 4 days. Cells were then fixed and stained with anti-CGN antibodies and DAPI.

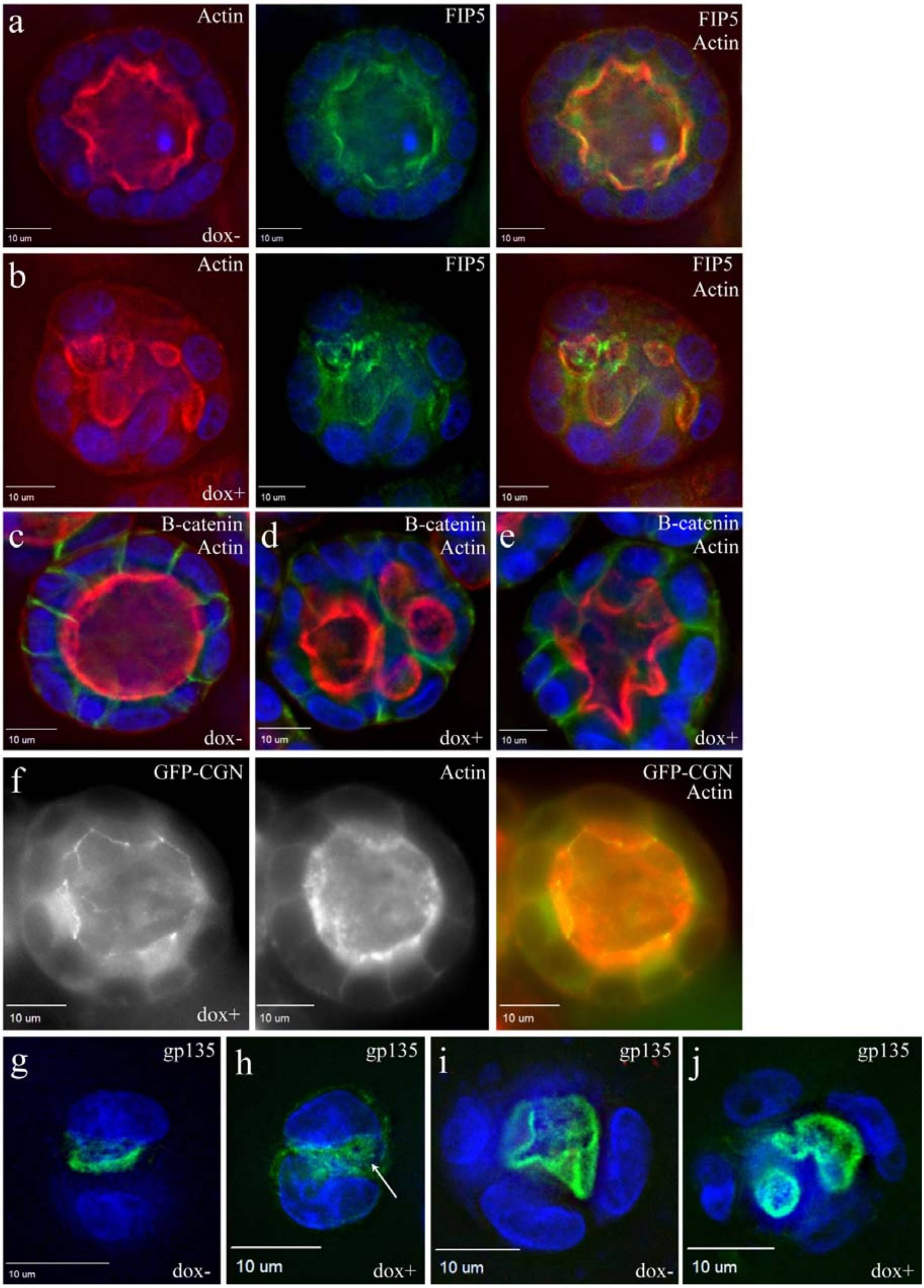

a
Actin
dox-
FIP5
FIP5
Actin
b
Actin
dox+
FIP5
FIP5
Actin
c
B-catenin
Actin
dox-
d
B-catenin
Actin
dox+
e
B-catenin
Actin
dox+
f
GFP-CGN
dox+
Actin
GFP-CGN
Actin
10 um
g
gp135
dox-
h
gp135
dox+
i
gp135
dox-
j
gp135
dox+
10 um

Supplementary Figure 2. CGN knock-down affects apical lumen formation

(a-b) MDCK cells stably expressing tet-inducible CGN shRNA were grown in the absence (a) or presence (b) of doxycycline for 4 days. Cells were then fixed and stained with phalloidin-Alexa594 (red) and FIP5 (green). (c-e) MDCK cells stably expressing tet-inducible CGN shRNA were grown in the absence (c) or presence (d,e) of doxycycline for 4 days. Cells were then fixed and stained with phalloidin-Alexa594 (red) and anti-β-catenin (green). (f) MDCK cells stably expressing tet-inducible CGN shRNA were transfected with human GFP-CGN (green) and grown in the presence of doxycycline for 4 days. Cells were then fixed and stained with phalloidin-Alexa594 (red). (g-j) MDCK cells stably expressing tet-inducible CGN shRNA were grown in the absence (g,i) or presence (h,j) of doxycycline for either 12 hours (g,h) or 3 days (i,j). Cells were then fixed and stained with anti-gp135 antibodies. Arrow in (h) points to ectopic lumen. Consecutive images without an individual letter label show the same cell.

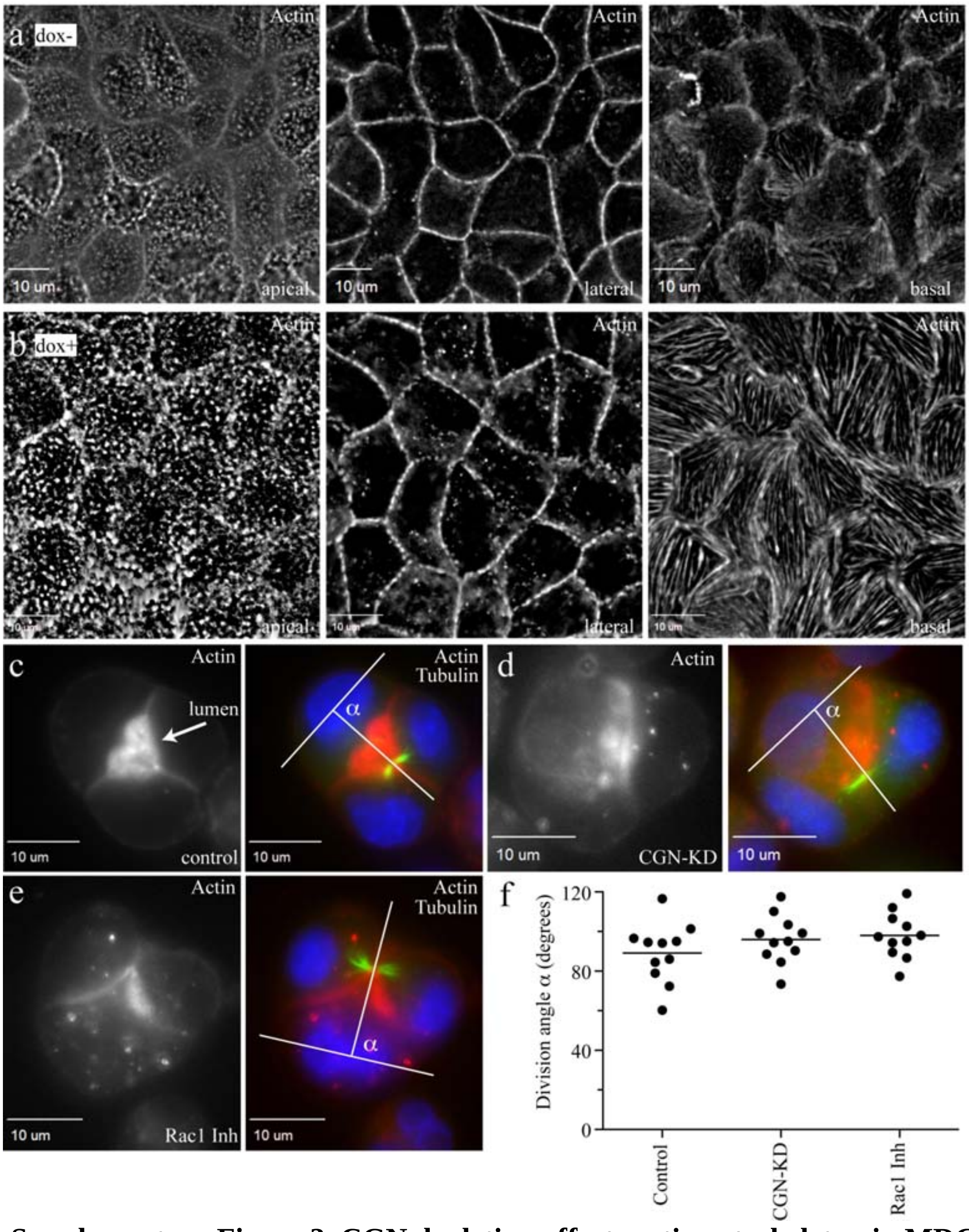

Supplementary Figure 3. CGN depletion affects actin cytoskeleton in MDCK cells

(a-b) MDCK cells stably expressing tet-inducible CGN shRNA were grown on Transwell filters in the presence (b) or absence (a) of doxycycline for 4 days. Cells were then fixed and stained with phalloidin-Alexa594 (red). Shown images are taken at apical (left images), lateral (mid-images) and basal (right images) poles of polarized MDCK cells.

(c-f) MDCK cells stably expressing tet-inducible CGN shRNA were embedded in Matrigel and grown for 24 hours in the presence (d) or absence (c and e) of doxycycline. During 24 hour incubation majority of cells undergo either 1 or two cell divisions, thus generating 3 or 4 cell organoids. Where indicated (e) cells were treated with Rac1 inhibitor for the first 12 hours

(during first cell division). Images marked with the same letter/number show the same cell. To analyze the second cell division angle, cell organoids were randomly chosen and α angle measured between long axis of interphase cell and division plane of cells in late cytokinesis (lines in c-e). Panel (f) shows quantitation of α angle from cells either depleted of CGN or treated with Rac1 inhibitor.

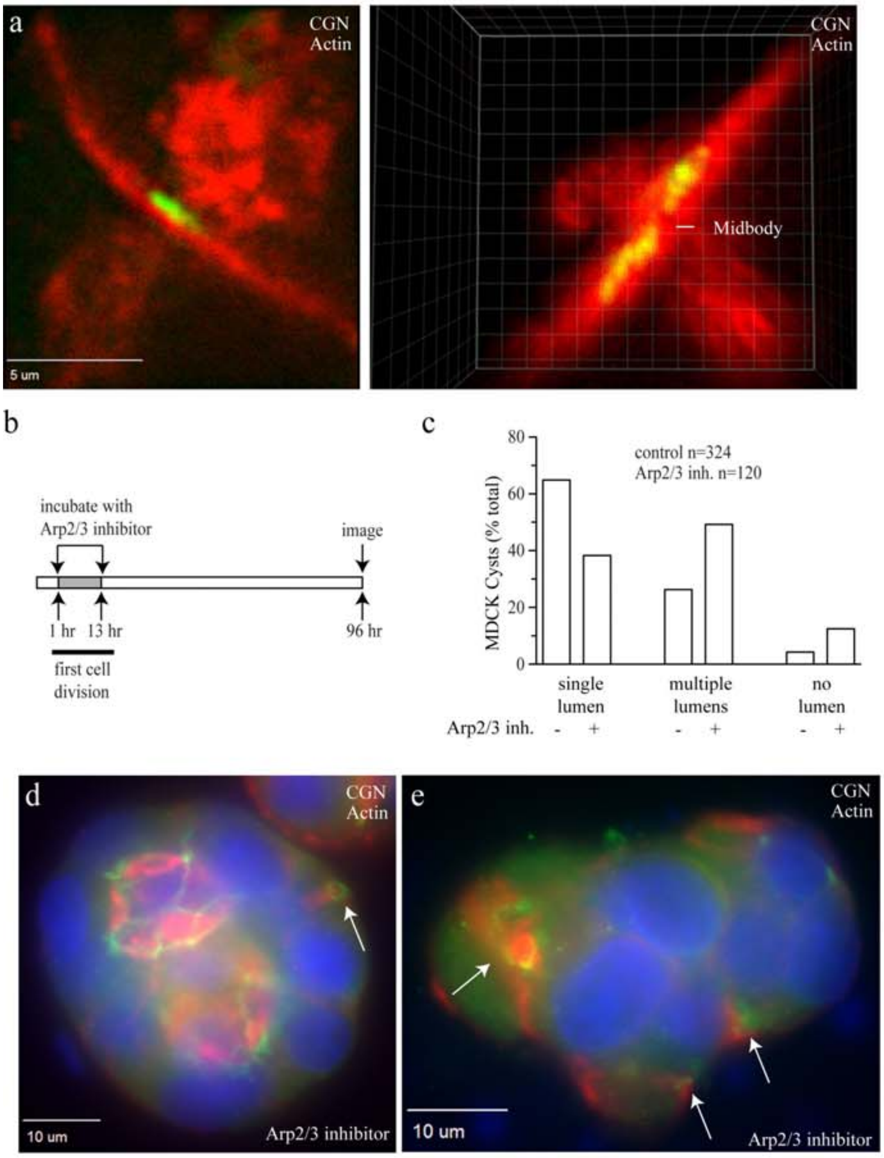

a
CGN
Actin
5 um
CGN
Actin
Midbody
b
incubate with
Arp2/3 inhibitor
image
1 hr
13 hr
96 hr
first cell
division
c
control n=324
Arp2/3 inh. n=120
MDCK Cysts (% total)
80
60
40
20
0
single
lumen
multiple
lumens
no
lumen
Arp2/3 inh.
-
+
-
+
-
+
d
CGN
Actin
10 um
Arp2/3 inhibitor
e
CGN
Actin
10 um
Arp2/3 inhibitor

Supplementary Figure 4. Midbody and Cingulin associated actin flares

(a) 3D-rendering images of midbody regions stained with phalloidin-Alexa594 (red) and anti-CGN (green) antibodies. Images shown are derived from cells depicted in Figure 3g.

(b) Schematic representation showing timing of 12-hour treatment with Arp2/3 inhibitor CK-666.

(c-e) MDCK cells were embedded in 3D Matrigel and allowed to grow for 24 hours. Where indicated, cells were treated with 200 µM Arp2/3 inhibitor CK-666 (d-e). Cells were then fixed and stained with phalloidin-Alexa594 (red) and anti-CGN (green) antibodies. Panel (c) shows quantitation of cysts with single lumen, multiple lumens or no lumen. n indicates number of cysts analyzed. Arrows point to ectopic CGN accumulations that associate with actin cytoskeleton.

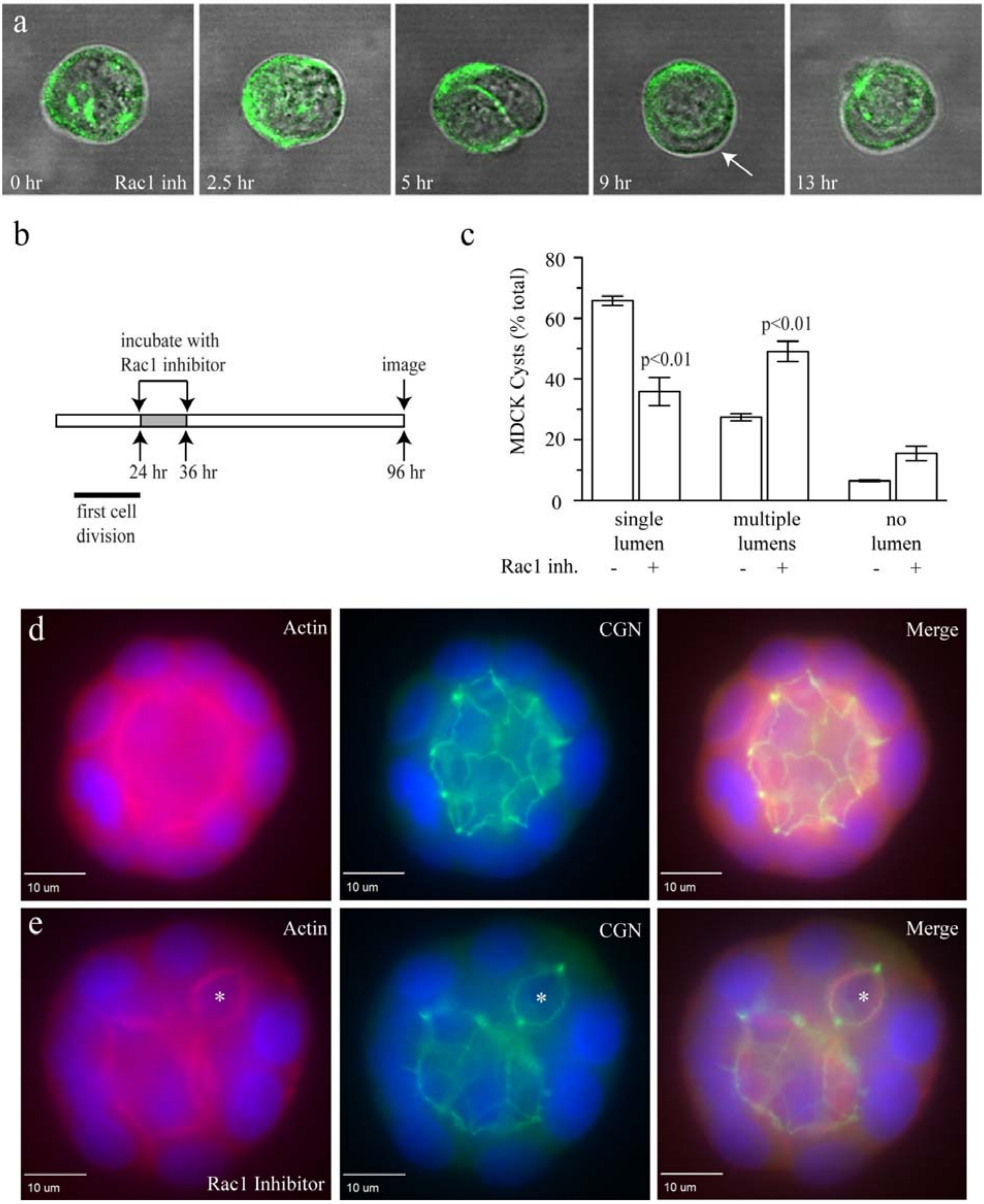

Supplementary Figure 5. Rac1 is required for apical lumen formation

(a) MDCK cells stably expressing GFP-CGN were embedded in 3D Matrigel and incubated in the presence of Rac1 inhibitor. Cells were then imaged using time-lapse microscopy.
(b) Schematic representation showing timing of 12-hour treatment with Rac1 inhibitor.
(c-e) MDCK cells were embedded in 3D Matrigel and allowed to grow for 4 days in the presence (e) or absence (d) of Rac1 inhibitor. The timing of inhibitor addition is shown in panel (b). Cells were then fixed and stained with phalloidin-Alexa594 (red) and anti-CGN (green) antibodies. Asterisks show smaller secondary lumen within the same cyst. Consecutive images without an individual letter label show the same cell. Panel (c) shows quantification of cells with single, multiple, or no lumens. Data shown are the means and standard deviations derived from three independent experiments.

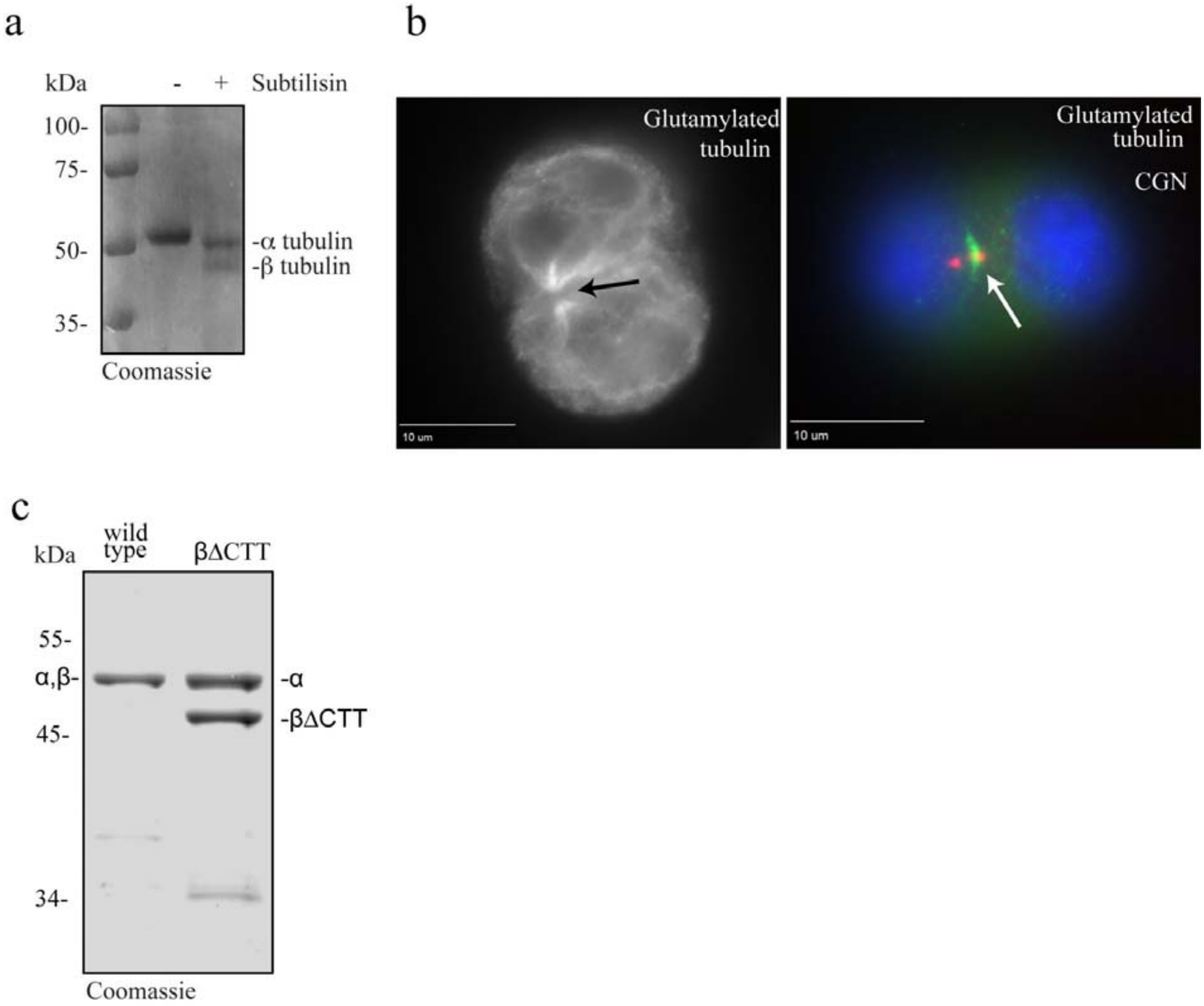

Supplementary Figure 6. CGN binds to tubulin C-terminal tail domains

(a) Untreated or subtilisin-treated mammalian tubulin was separated on SDS/PAGE gels and subjected to Coomassie staining. Note decrease in α-tubulin and β-tubulin size in subtilisin-treated samples due to the removal of CTTs.

(b) MDCK cells were embedded in 3D Matrigel and allowed to grow for 24 hours. Cells were then fixed and stained with anti-glutamylated tubulin (red) and anti-CGN (green) antibodies. Black arrow points to midbody, white arrow to the AMIS.

(c) Tubulin purified from wild-type or β-tubulin (TUB2) mutant yeast. Note the decrease in β-tubulin size in mutant strain due to the removal of CTT.

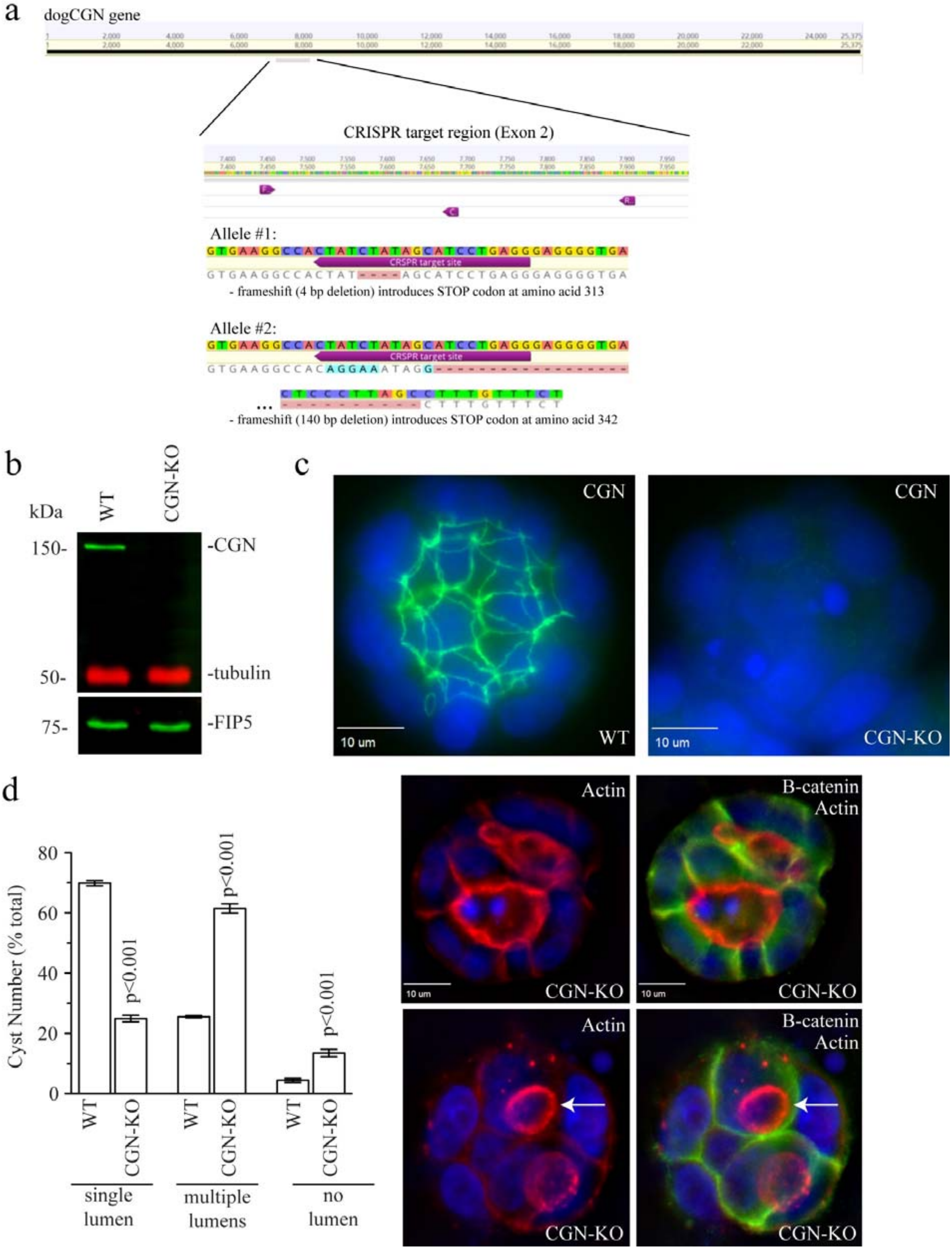

a
dogCGN gene
CRISPR target region (Exon 2)
Allele #1:
GTGAAGGCCACTATCTATAGCATCCTGAGGGAGGGGTGA
CRSPR target site
GTGAAGGCCACTAT----AGCATCCTGAGGGAGGGGTGA
- frameshift (4 bp deletion) introduces STOP codon at amino acid 313
Allele #2:
CRSPR target site
- frameshift (140 bp deletion) introduces STOP codon at amino acid 342
b
kDa
WT
CGN-KO
150-
50-
75-
-CGN
-tubulin
-FIP5
c
CGN
WT
CGN-KO
10 um
d
Cyst Number (% total)
80
60
40
20
0
p<0.001
WT
CGN-KO
single lumen
multiple lumens
no lumen
Actin
B-catenin Actin

Supplementary Figure 7. Generation of CGN knock-out in MDCK cells using CRISPR/Cas9

(a) Schematic representation of CRISPR guide sequence designed to target canine CGN in MDCK cells. Arrows marked with “F” and “R” shows the location of primers used to amplify and sequence the region targeted with CRISPR/Cas9. Allele#1 and allele#2 show the mutation in CGN exon#2 that were introduced in both CGN genes.

(b) Western blot analysis comparing CGN levels in cellular lysates isolated from a parental MDCK cells (WT) or MDCK cells co-expressing CGN CRISPR guide and Cas9 (CGN-KO).

(c) Comparison of CGN levels in 4 day-old epithelial cysts formed by either parental MDCK cells (WT) or MDCK cells co-expressing CGN CRISPR guide and Cas9 (CGN-KO).

(d) Parental MDCK cells (WT) or MDCK cells co-expressing CGN CRISPR guide and Cas9 (CGN-KO) were embedded in 3D Matrigel and grown for 4 days. Cells were then fixed and stained with phalloidin-Alexa596 (red) and anti-β-catenin antibodies (green). Arrows point to ectopic muni-lumen forming inside the cell. Quantification of the number of cysts with either single, multiple or no lumens are shown in left panel. Data shown are the means and standard deviations derived from three independent experiments.

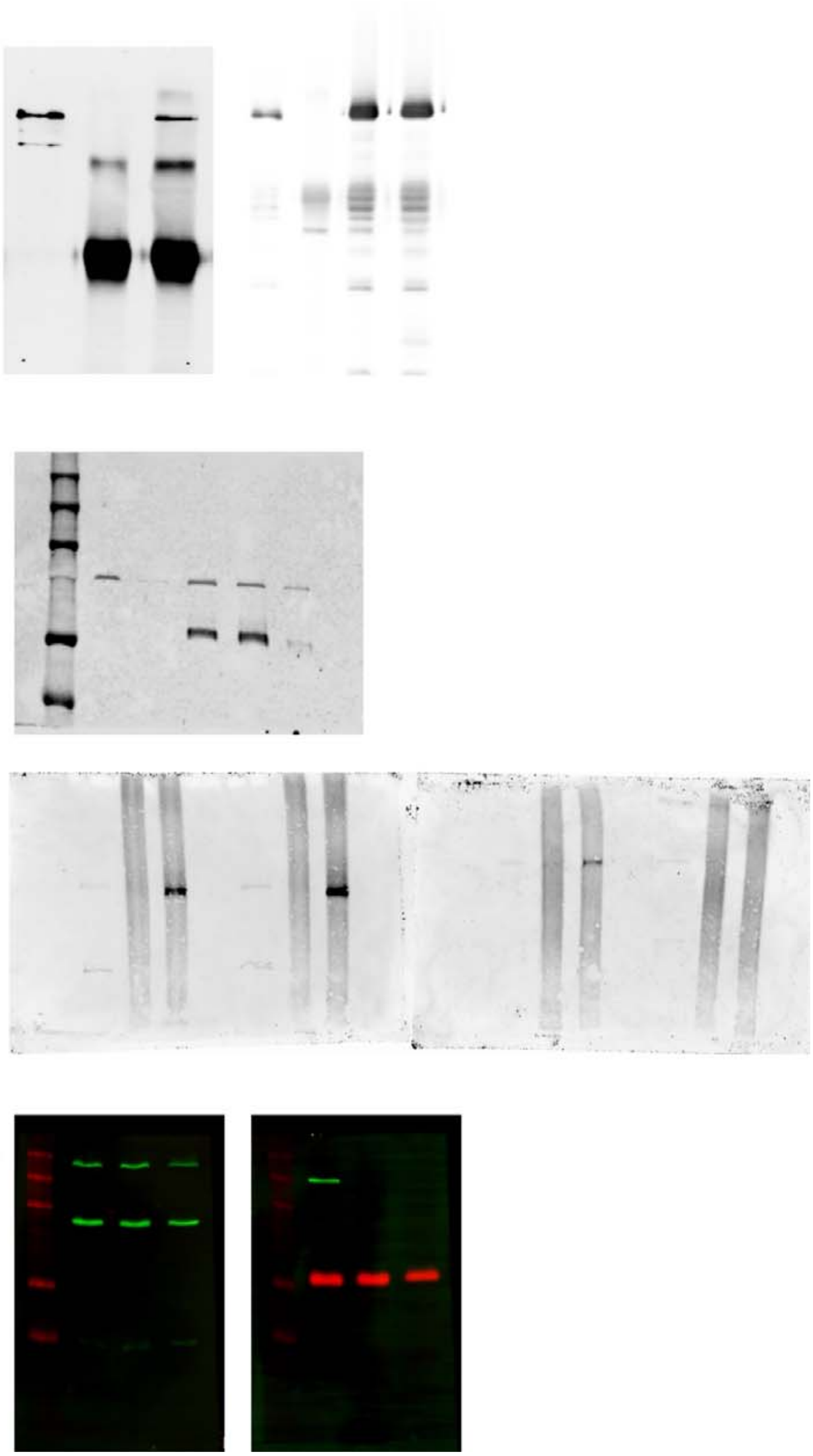

a

b

c

d

Supplementary Figure 8. Uncropped scans of western blots

(a) Figure 1d.

(b) Figure 8e.

(c) Supplemental Figure 1b.

(d) Supplemental Figure 7b.
